# In-silico assessment of high-risk non-synonymous SNPs in ADAMTS3 gene associated with Hennekam syndrome and their impact on protein stability and function

**DOI:** 10.1186/s12859-023-05361-6

**Published:** 2023-06-15

**Authors:** Khyber Shinwari, Yurong Wu, Hafiz Muzzammel Rehman, Ningkun Xiao, Mikhail Bolkov, Irina Tuzankina, Valery Chereshnev

**Affiliations:** 1 Institute of Chemical Engineering, Department of Immunochemistry, Ural Federal University, Yekaterinburg, Russia; 2 Department of Chemistry, Hong Kong University of Science and Technology, Hong Kong, China; 3 University of the Punjab Institute of Biochemistry and Biotechnology, Lahore, Pakistan; 4 Department of Psychology, Ural Federal University, Yekaterinburg, Russia; 5 Insitutite of Immunology and Physiology, Russian Academy of Science, Yekaterinburg, Russia

**Keywords:** Primary immunodeficiency, Hennekam syndrome, In-silico, *ADAMTS3*, Nonsynonymous SNP

## Abstract

Hennekam Lymphangiectasia–Lymphedema Syndrome 3 (HKLLS3) is a rare genetical disorder caused by mutations in a few genes including ADAMTS3. It is characterized by lymphatic dysplasia, intestinal lymphangiectasia, severe lymphedema and distinctive facial appearance. Up till now, no extensive studies have been conducted to elucidate the mechanism of the disease caused by various mutations. As a preliminary investigation of HKLLS3, we sorted out the most deleterious nonsynonymous single nucleotide polymorphisms (nsSNPs) that might affect the structure and function of ADAMTS3 protein by using a variety of in silico tools. A total of 919 nsSNPs in the ADAMTS3 gene were identified. 50 nsSNPs were predicted to be deleterious by multiple computational tools. 5 nsSNPs (G298R, C567Y, A370T, C567R and G374S) were found to be the most dangerous and can be associated with the disease as predicted by different bioinformatics tools. Modelling of the protein shows it can be divided into segments 1, 2 and 3, which are connected by short loops. Segment 3 mainly consists of loops without substantial secondary structures. With prediction tools and molecular dynamics simulation, some SNPs were found to significantly destabilize the protein structure and disrupt the secondary structures, especially in segment 2. The deleterious effects of mutations in segment 1 are possibly not from destabilization but from other factors such as the change in phosphorylation as suggested by post-translational modification (PTM) studies. This is the first-ever study of ADAMTS3 gene polymorphism, and the predicted nsSNPs in ADAMST3, some of which have not been reported yet in patients, will serve for diagnostic purposes and further therapeutic implications in Hennekam syndrome, contributing to better diagnosis and treatment.

**Supplementary Information:**

The online version contains supplementary material available at 10.1186/s12859-023-05361-6.

## Introduction

Hennekam syndrome is a rare autosomal recessive disorder that manifests with abnormalities in the lymphatic system. It was first described by Dutch physician Hennekam in 1989 [1]. Despite only approximately 50 documented cases of this syndrome in the literature [2], it represents a significant threat to patients' health and quality of life due to its characteristic symptoms, including lymphedema, lymphangiectasia, facial anomalies, and cognitive impairment [3, 4]. Thus, understanding the pathogenic mechanisms underlying Hennekam syndrome is critical to mitigating its detrimental effects on patients.

Approximately 25% of individuals with Hennekam syndrome display homozygous variations in the CCBE1 gene (Collagen and Calcium Binding EGF Domains 1) [5], which can include pathogenic variations such as missense, splicing, small insertions, and deletions [4]. CCBE1 is a protein-coding gene that plays a role in various biological processes, including angiogenesis, lymph angiogenesis, and cell adhesion and is implicated in regulating the lymphatic system. Another protein-coding gene associated with Van Maldergem Syndrome 2 and Hennekam Lymphangiectasia–Lymphedema Syndrome 2 is the FAT4 gene (FAT Atypical Cadherin 4), which is involved in approximately 20% of Hennekam syndrome cases [6]. In 2017, a study reported two children with bi-allelic missense mutations in the ADAMTS3 gene (L168P and I291T) [6]. These mutations are located within the prodomain region of the ADAMTS3 protein, which regulates enzyme activation but is not situated within the active site of the ADAMTS3 protein. Half of all cases involve mutations in one or more of these genes, while the etiology of the remaining cases is unknown. Mutations in the CCBE1 gene have been associated with Aagenaes (cholestasis-lymphedema) syndrome, and biallelic mutations in the FAT4 gene can cause Van Maldergem syndrome type 2 [7].

Furthermore, it has been established that ADAMTS3, which is predominantly expressed in cartilage, is involved in skin, lung, and aorta pathologies, as well as being colocalized with type II pro-collagen, a crucial cartilage component, and the nervous system. ADAMTS3 is also more abundant in human skin and skin fibroblasts compared to ADAMTS2 [8]. Procollagen N proteinases, including ADAMTS2, ADAMTS3, and ADAMTS14, play roles in collagen biosynthesis, maturation, inflammation, and wound healing [9–11]. ADAMTS3 associates with CCBE1 for VEGF-C activation, and the primary axis regulating lymphangiogenesis involves the VEGFR3 receptor (encoded by FLT4) and its ligand, VEGF-C. Mutations in this pathway may disrupt regulatory mechanisms.

Hence, the identification of mutated genes and their functions has become the focus of our attention. Single nucleotide polymorphisms (SNPs) occur every 200–300 base pairs in the human genome, serving as genetic markers [12]. Approximately 0.5 million SNPs are present within the coding region of the genome [13]. Amino acid substitutions in conserved domains can influence the structure, stability, and function of proteins. Nonsynonymous SNPs (nsSNPs) can alter protein function, leading to an increased risk of human diseases [14–16]. Studies have demonstrated that nsSNPs account for 50% of the variants associated with inheritable genetic diseases [17–19]. Various advanced tools employ alignment techniques based on matrix and data tree structure calculations [13, 20, 21]. Bioinformatics plays a critical role in predicting deleterious SNPs associated with human genetic disorders. In this study, we utilized computational tools and algorithms to analyze vast amounts of genetic data, identify candidate ADAMST3 SNPs, and predict their functional impact on protein structure and function in Hennekam syndrome. This in silico approach facilitates the prioritization of potentially pathogenic variants, significantly reducing the time and resources required for experimental validation. Moreover, we anticipate that this study will facilitate the comprehension of the underlying mechanisms, diagnosis, and targeted therapy design for Hennekam syndrome. This contribution will advance personalized medicine and aid in the management of genetic disorders in humans.

## Results

### dbSNPs/NCBI investigation of the desired gene

**Fig. 1:**
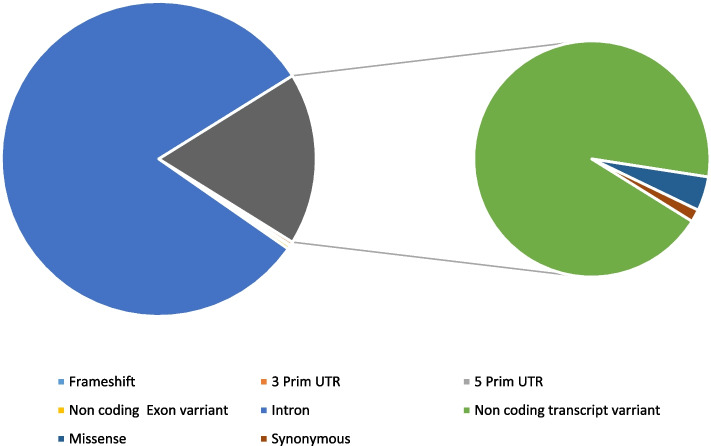
Distribution of SNPs present in the ADAMTS3 gene

**Fig. 2:**
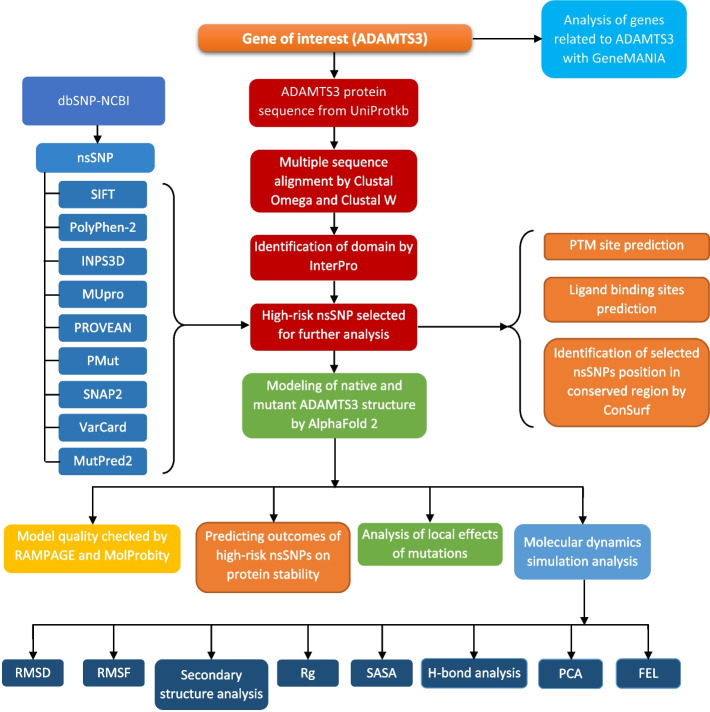
Flowchart of methodology

The SNPs of the ADAMTS3 gene were examined using the NCBI database (http://www.ncbi.nlm.nih.gov/) and the Ensembl genome browser (http://asia.ensembl.org/Homo_sapiens/Gene/Summary). Figure 1 illustrates that a total of 70,876 SNPs have been identified in Homo sapiens, of which 848 are nonsynonymous (missense) SNPs in the NCBI database. In Ensembl, 919 nsSNPs and 325 synonymous SNPs were found. The methodology for ADAMTS3 gene analysis is summarized in Fig. 2.

### Analysis of genes related to ADAMTS3 with GeneMANIA

**Fig. 3:**
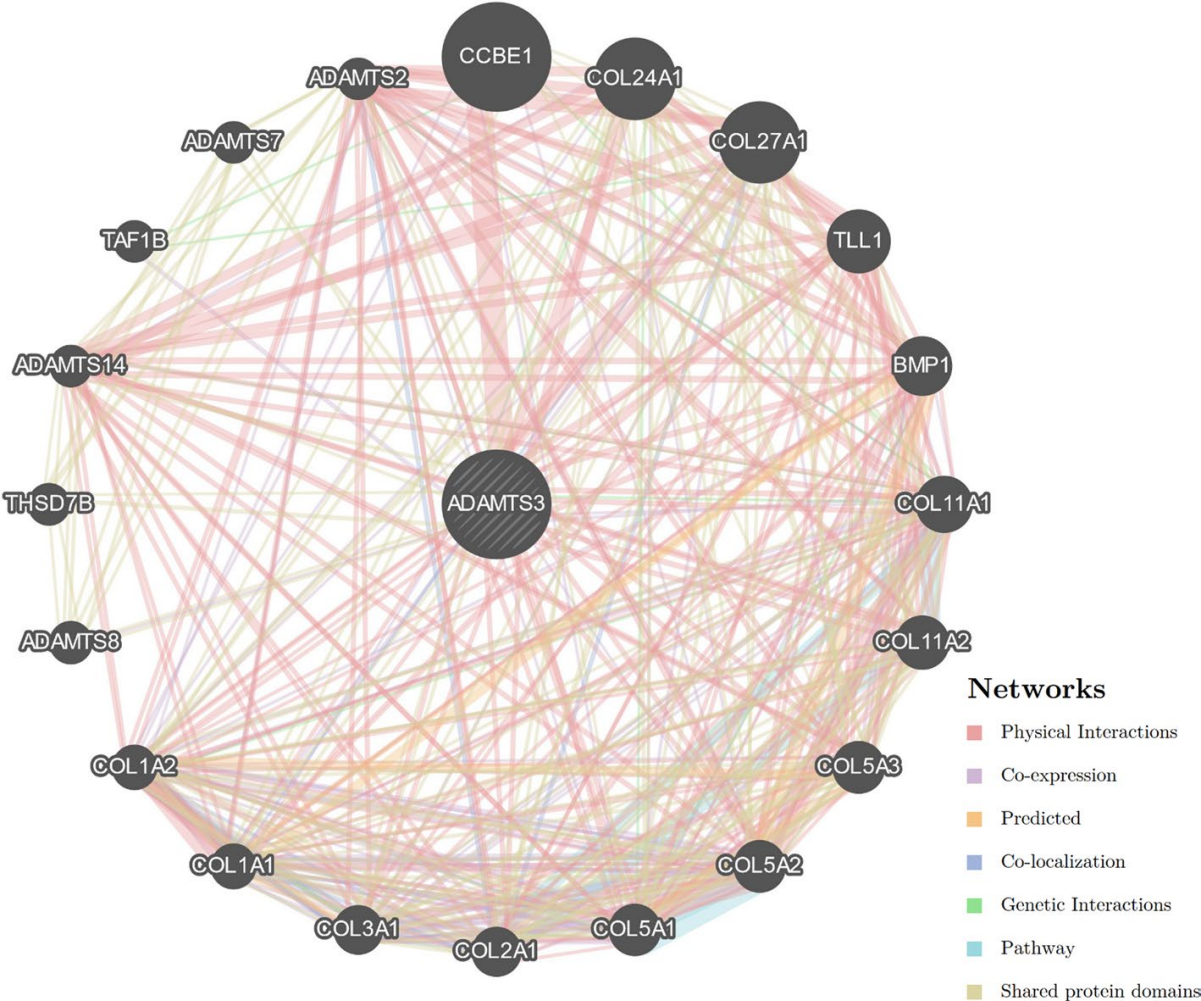
Genes related to ADAMTS3

According to our analysis, the ADAMTS3 gene is related to 20 other genes, including CCBE1, COL24A1, COL27A1, TLL1, BMP1, COL11A1, COL11A2, COL5A3, COL5A2, COL5A1, COL2A1, COL3A1, COL1A1, COL1A2, ADAMTS8, THSD7B, ADAMTS14, TAF1B, ADAMTS7 and ADAMTS2. Among these, five genes—COL11A1, COL5A2, COL1A1, ADAMTS8 and TAF1B—are co-expressed with ADAMTS3. Additionally, ADAMTS3 shares a common domain with five genes: ADAMTS2, ADAMTS7, ADAMTS8, ADAMTS14 and THSD7B (refer to Fig. 3 and Table 1 for details).

**Table 1:** Genes co-expressed and sharing a domain with *ADAMTS3*

Gene symbol	Description	Co-expression	Shared domain
CCBE1	Collagen and calcium binding EGF domains 1	No	No
COL24A1	Collagen type XXIV alpha 1 chain	No	No
COL27A1	Collagen type XXVII alpha 1 chain	No	No
TLL1	Tolloid like 1	No	No
BMP1	Bone morphogenetic protein 1	No	No
COL11A1	Collagen type XI alpha 1 chain	Yes	No
COL11A2	Collagen type XI alpha 2 chain	No	No
COL5A3	Collagen type V alpha 3 chain	No	No
COL5A2	Collagen type V alpha 2 chain	Yes	No
COL5A1	Collagen type V alpha 1 chain	No	No
COL2A1	Collagen type II alpha 1 chain	No	No
COL3A1	Collagen type III alpha 1 chain	No	No
COL1A1	Collagen type I alpha 1 chain	Yes	No
COL1A2	Collagen type I alpha 2 chain	No	No
ADAMTS8	ADAM metallopeptidase with thrombospondin type 1 motif 8	Yes	Yes
THSD7B	Thrombospondin type 1 domain containing 7B	No	Yes
ADAMTS14	ADAM metallopeptidase with thrombospondin type 1 motif 14	No	Yes
TAF1B	TATA-box binding protein associated factor, RNA polymerase I subunit B	Yes	No
ADAMTS7	ADAM metallopeptidase with thrombospondin type 1 motif 7	No	Yes
ADAMTS2	ADAM metallopeptidase with thrombospondin type 1 motif 2	No	Yes

These findings suggest that ADAMTS3 may be functionally related to the co-expressed genes and may share common biological pathways. Furthermore, the shared domain between ADAMTS3, THSD7B and other protein family members of ADAMTS suggests that they may have similar protein structures and potentially similar functions. These results provide a basis for further investigation into the biological roles of ADAMTS3 and its potential involvement in related pathways.

### SIFT and PolyPhen-2’s prediction of deleterious nsSNPs in ADAMTS3

In this study, we examined a total of 919 nonsynonymous SNPs (nsSNPs) to determine their potential impacts on protein structure and function. Among the 919 nsSNPs, SIFT and PolyPhen-2 predicted 45 (forty-five) to be deleterious or damaging, as shown in Table 2. Additionally, SIFT predicted 50 nsSNPs to be potentially damaging. It is worth noting that minor allele frequency (MAF) information was available for only sixteen nsSNPs. For other nsSNPs, the MAFs may be less than 1%. These results suggest that a small proportion of nsSNPs within the ADAMTS3 gene may have deleterious or damaging effects on the encoded protein, potentially contributing to disease susceptibility or progression. Further investigation into the functional consequences of these nsSNPs is warranted to better understand their potential roles in disease etiology.

**Table 2:** SIFT and PolyPhen-2 predicted non-synonymous SNPs

No	ID of nsSNPs	AA positions	SIFT	Score	PolyPhen-2	Score	No	ID of nsSNPs	AA positions	SIFT	Score	PolyPhen-2	Score
1	rs61757480	I291T	DEL	0.001	Prob Dam	1	26	rs370857003	S58F	DEL	0	Prob Dam	1
2	rs61741624	V395I	DEL	0.035	Prob Dam	1	27	rs370665830	Q588H	DEL	0.007	Prob Dam	0.979
3	rs140806973	A336V	DEL	0.035	Prob Dam	1	28	rs143125991	G25V	DEL	0.016	Prob Dam	0.982
4	rs80330149	G298R	DEL	0.004	Prob Dam	1	29	rs146979323	R565W	DEL	0	Prob Dam	1
5	rs137870001	R270H	DEL	0.02	Prob Dam	1	30	rs372028526	R248H	DEL	0.002	Prob Dam	1
6	rs138927279	C567Y	DEL	0	Prob Dam	1	31	rs372067284	T668M	DEL	0.001	Prob Dam	1
7	rs139835107	F81L	DEL	0.019	BENIGN	0.416	32	rs372614431	R817C	DEL	0.001	Prob Dam	0.995
8	rs140595148	Q616H	DEL	0	Prob Dam	1	33	rs372664222	R713L	DEL	0.004	Prob Dam	0.998
9	rs140914273	R572H	DEL	0.017	Prob Dam	1	34	rs374592996	Y148C	DEL	0.001	Prob Dam	0.999
10	rs141374503	R565Q	DEL	0.007	Prob Dam	1	35	rs374628791	R55L	DEL	0.018	BENIGN	0.223
11	rs142268705	A370T	DEL	0.002	Prob Dam	1	36	rs374705972	R435H	DEL	0.029	Prob Dam	1
12	rs142781084	Q927R	DEL	0.017	Prob Dam	1	37	rs374267184	I287F	DEL	0.001	Prob Dam	1
13	rs143059623	Y536C	DEL	0.003	Prob Dam	0.999	38	rs147882384	N98S	DEL	0.012	Prob Dam	0.999
14	rs144367585	M731T	DEL	0.009	Possibly Dam	0.464	39	rs374979020	R883C	DEL	0	Prob Dam	1
15	rs145610471	F777L	DEL	0.01	Prob Dam	0.921	40	rs375640530	Y636C	DEL	0.011	Prob Dam	0.996
16	rs141331151	P371S	DEL	0.01	Prob Dam	0.994	41	rs375983592	R959W	DEL	0	Prob Dam	1
17	rs139921635	P513T	DEL	0.01	Prob Dam	1	42	rs377163219	R576L	DEL	0	Prob Dam	1
18	rs149118364	R943H	DEL	0.03	BENIGN	0.067	43	rs377294345	R954H	DEL	0.02	Prob Dam	1
19	rs150168072	R572C	DEL	0.001	Prob Dam	1	44	rs201820778	R1053C	DEL	0.038	Prob Dam	1
20	rs201524333	S1038F	DEL	0.021	BENIGN	0.368	45	rs374977549	P510A	DEL	0.054	Prob Dam	1
21	rs202031187	G374S	DEL	0.005	Prob Dam	1	46	rs200161568	D791V	DEL	0.021	Prob Dam	0.908
22	rs202136117	D815Y	DEL	0.008	Prob Dam	0.767	47	rs188256169	P77T	DEL	0.022	BENIGN	0.003
23	rs202230275	R94Q	DEL	0.008	Prob Dam	0.897	48	rs150652042	R137W	DEL	0.032	BENIGN	0
24	rs367831484	G983S	DEL	0.02	Prob Dam	1	49	rs150012152	G412S	DEL	0.02	Prob Dam	1
25	rs368865428	C567R	DEL	0	Prob Dam	1	50	rs149726955	L801F	DEL	0.004	Prob Dam	1

Threshold: SIFT: < 0.05 PolyPhen-2: Probably Damaging: score > 0.908; Possibly Damaging: 0.446 < score ≤ 0.908; Benign: score ≤ 0.446

### Confirmatory analysis of the deleterious nsSNPs through different mutation prediction tools

Various computational algorithms were employed to investigate the pathogenic implications of the 919 nsSNPs in the ADAMTS3 gene. Out of these, only 50 nsSNPs were identified as completely deleterious by the SIFT tool and subjected to further analysis by several other tools. The top 5 nsSNPs were determined to be deleterious by all algorithmic tools. However, the number of deleterious SNPs varied among the different computational tools. For instance, PolyPhen-2 predicted 45 deleterious nsSNPs but did not validate any of the five nsSNPs that SIFT classified as deleterious with a cutoff of > 0.5. The FATHMM tool identified only 10 nsSNPs (20%) out of 50 SIFT-predicted nsSNPs as deleterious, while SNAP2 predicted 45 nsSNPs (90%) as harmful, and PROVEAN predicted 45 nsSNPs (90%) to be severely detrimental. The PANTHER algorithm predicted the deleterious impact of 46 (92%) nsSNPs, with 40 nsSNPs classified as probably damaging, 6 nsSNPs as possibly damaging, and 4 as probably benign on the ADAMTS3 protein. Mutation Assessor identified 12 nsSNPs as deleterious, while P-Mut predicted 25 (65.21%) to be deleterious. LRT predicted 44 (88%) nsSNPs to be deleterious, while PhD-SNP [22] and PON-P2 revealed 26 (52%) deleterious SNPs. Similarly, SNPs&GO and M-CAP showed 33 (66%) and 39 (89%) nsSNPs as deleterious, respectively. MetaLR and MetaSVM showed 18 (36%) and 22 (44%) nsSNPs as deleterious, respectively. A set of five nsSNPs were deemed to be extremely deleterious as they were fully supported by all current techniques. However, PhD-SNP [22] contradicted the G374S result obtained using other technologies. All prediction methods produced statistically significant results with a *p* value of 0.001. The results of mutation prediction tools other than SIFT and PolyPhen-2 and the significance of all other tools are presented in Additional file 1: File S1. Results of mutation prediction tools other than SIFT and PolyPhen-2 and the significance of all other tools are displayed (Table 3).

**Table 3:** Confirmation of highly deleterious nsSNPs by other prediction software

A.A position	PROVEAN	FATHMM	LRT	M-CAP	MetaSVM	MetaLR	Mutation Assessor	MutationTaster	FATHMM MKL Coding	CADD	PhD-SNP	PANTHER	SNPs&GO	PON-P2	DANN	SNAP2
G298R	D	D	D	D	D	D	D	D	D	D	D	D	D	D	D	D
C567Y	D	D	D	D	D	D	D	D	D	D	D	D	D	D	D	D
A370T	D	D	D	D	D	D	D	D	D	D	D	D	D	D	D	D
C567R	D	D	D	D	D	D	D	D	D	D	D	D	D	D	D	D
G374S	D	D	D	D	D	D	D	D	D	D	N	D	D	D	D	D
G983S	D	T	D	D	D	D	D	D	D	D	D	D	D	D	D	D
R435H	D	D	D	D	D	D	D	D	D	D	N	D	D	D	D	D
Q616H	D	T	D	D	D	D	D	D	D	D	D	D	D	D	D	D
I291T	D	T	D	D	D	D	D	D	D	D	D	D	D	D	D	D
T668M	D	T	D	D	D	D	D	D	D	D	D	D	D	D	D	D
R572C	D	T	D	D	D	T	D	D	D	D	D	D	D	D	D	D
R576L	D	T	D	D	D	D	D	D	D	D	D	D	D	D	D	D
S58F	D	T	D	D	D	D	D	D	D	D	D	D	N	D	D	D
R565W	D	T	D	D	D	T	D	D	D	D	D	D	D	D	D	D
A336V	D	D	D	D	D	D	D	D	D	D	N	D	N	D	D	D
R959W	D	T	D	D	D	D	D	D	D	D	N	D	D	D	D	D
G412S	D	D	D	D	D	D	T	D	D	D	N	D	D	D	D	D
P371S	D	D	D	D	D	D	D	D	D	D	N	D	D	U	D	D
R883C	D	T	D	D	D	T	D	D	D	D	N	D	D	D	D	D
Y636C	D	T	T	D	D	D	D	T	D	D	D	D	D	D	D	D
Y536C	D	T	D	D	T	T	D	D	D	D	D	D	D	D	D	D
R954H	D	T	D	D	D	T	D	D	D	D	D	D	D	U	D	D

### MutPred2 predictions of pathogenic amino acid substitutions

MutPred2 is a computational tool that evaluates the molecular properties of amino acid residues in humans to determine whether a substitution is associated with the disease. The score generated by MutPred2 indicates the likelihood of an amino acid substitution affecting the protein's function. A score threshold of 0.5 is used to predict pathogenicity, with scores greater than or equal to 0.8 considered highly confident [23]. The higher the score, the more probable it is that an amino acid substitution is linked to a particular disease. Additional file 2: File S2 displays the results of MutPred2.

### Conservation analysis

Based on the ConSurf analysis, it was found that 50 missense deleterious SNPs were distributed across various locations (with conservation scores of 7, 8 or 9) (see Additional file 3: File S3). Among these variants, 26 were located in highly conserved regions, with 19 of them (Q927R, G298R, C567Y, C567R, Q616H, R565W, R565Q, P371S, P513T, R248H, T668M, R435H, N98S, R883C, G412S, L801F, S1038F, G983S, R959W) are expected to be functional and exposed residues, while the remaining 7 (I291T, V395I, A336V, G374S, S58F, I287F, and A370T) were predicted to be buried and structural residues. The analysis also revealed several conserved and buried residues, including F81L, Y148C, R435H, Y536C, M731T, F777L, R94L, R270H, P510A, R572C, R572H, Q588H, R713L, R817C, R943H, and R954H. Additionally, eight residues were found to be exposed (G25V, R55L, P77T, R137W, Y636C, D791V, D815Y, and R1053C).

### Protein modeling

**Fig. 4:**
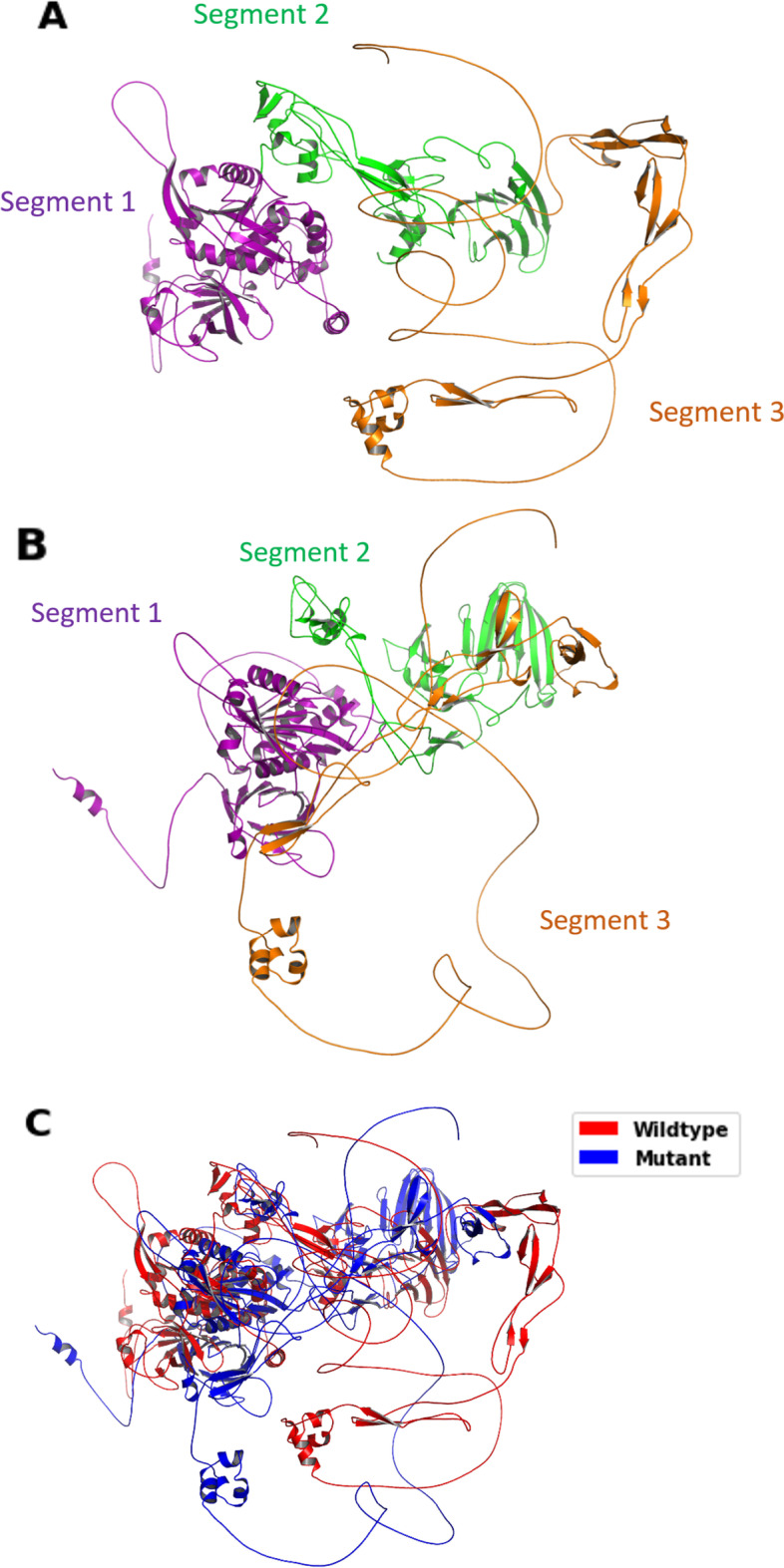
Chimera structure of ADAMTS3 protein. Wildtype (**A**) and mutant (**B**) structures of segment 1 (residues 1–466), segment 2 (residues 467–831) and segment 3 (residues 832–1205). These three segments are connected by loops. Segment 3 mainly consists of loops. **C** Superposition (alignment) of wildtype (red) and mutant (blue) structures

The wildtype and mutant ADAMT3 structures are predicted by AlphaFold 2. 25 mutations are incorporated in the modeling of the mutant structure. 21 of them (S58F, I291T, G298R, A336V, A370T, P371S, G374S, G412S, R435H, Y536C, R565W, C567R, R572C, R576L, Q616H, Y636C, T668M, R883C, R954H, R959W and G983S) are confirmed by multiple software as deleterious (C567Y is not included because it occupies the same position as C567R), while 4 of them (R138K, R574C, C578L and Q606H) are discovered clinically. The wildtype and mutant models are validated by Ramachandran plot and MolProbity all-atom contact analysis (see Methods). The wildtype model shows 1032 residues (85.8%) in the favored region, 77 (6.4%) in the allowed region and 94 (7.8%) in the outlier region, and the total residues in the favored and the allowed regions are 1109 (92.2%). The mutant model shows 1008 residues (83.8%) in the favored region, 110 (9.1%) in the allowed region and 85 (7.1%) in the outlier region. and the total residues in the favored and the allowed regions are 1118 (92.9%). (Additional file 4: File S4) For MolProbity all-atom contact analysis, the wildtype protein shows a clashscore of 3.61 while the mutant one shows 1.88 (Additional file 5: File S5), which are acceptable values. The structures can be divided into 3 segments (segment 1: Met1-Pro466; segment 2: Lys467-Val831; segment 3: Pro832-Arg1205), which are connected by loops (Fig. 4A, B). Segment 3 of both proteins mainly consists of loops without many secondary structures, so we consider it as an inaccurate prediction and ignore it for further analysis. We mainly focus on segments 1 and 2, which contain extensive secondary structures. We assume that there are insignificant interactions between segments, so mutations in one segment will not substantially affect another one. The superposition of the wildtype and mutant structures (Fig. 4C) shows an RMSD value of 30.367 Å.

### Predicting outcomes of high-risk nsSNPs on protein stability

The impact of 50 high-risk nonsynonymous single nucleotide polymorphisms (nsSNPs) in ADAMTS3 on protein stability was evaluated using MUpro and INPS3D (Additional file 6: File S6). The MUpro analysis predicted that 47 nsSNPs would decrease protein stability (ΔΔG < 0), while 3 nsSNPs would increase it (ΔΔG > 0). The INPS3D analysis identified 44 nsSNPs that individually decreased protein stability. Although a majority of the nsSNPs were predicted to have destabilizing effects, G412S, R576L, and R954H (identified by MUpro), and G25V, R55L, S58F, R572C, R713L, and D791V (identified by INPS3D) were found to enhance protein stability by both methods. However, the variants F81L, Y148C, I287F, I291T, A370T, Y536C, C567R, C567Y, and M731T were predicted to disrupt the protein structure and function, with ΔΔG values less than − 1 kcal/mol, as determined by both tools.

### High-risk nsSNPs consequences on ligand binding sites prediction

The ligand-binding sites of the ADAMTS3 protein were predicted using the RaptorX Binding server (pocket multiplicity value greater than 40) and the COACH ligand-binding site prediction server. The RaptorX Binding analysis identified a pocket multiplicity of 151, which was the highest observed, and was linked to the residues G365, M366, Q367, G368, Y369, V395, H398, E399, H402, H408, A426, P427, L428, and V429, with an expected Zn^2+^ cation ligand. The COACH server predicted a Zn^2+^ cation binding site with a C-score of 0.15, located at residues H398, H402, and H408. The rank 2 sites identified by COACH were linked to a Co^2+^ cation at residues E259, L334, 351, 355, and 356.

### Local effects of mutations

**Fig. 5:**
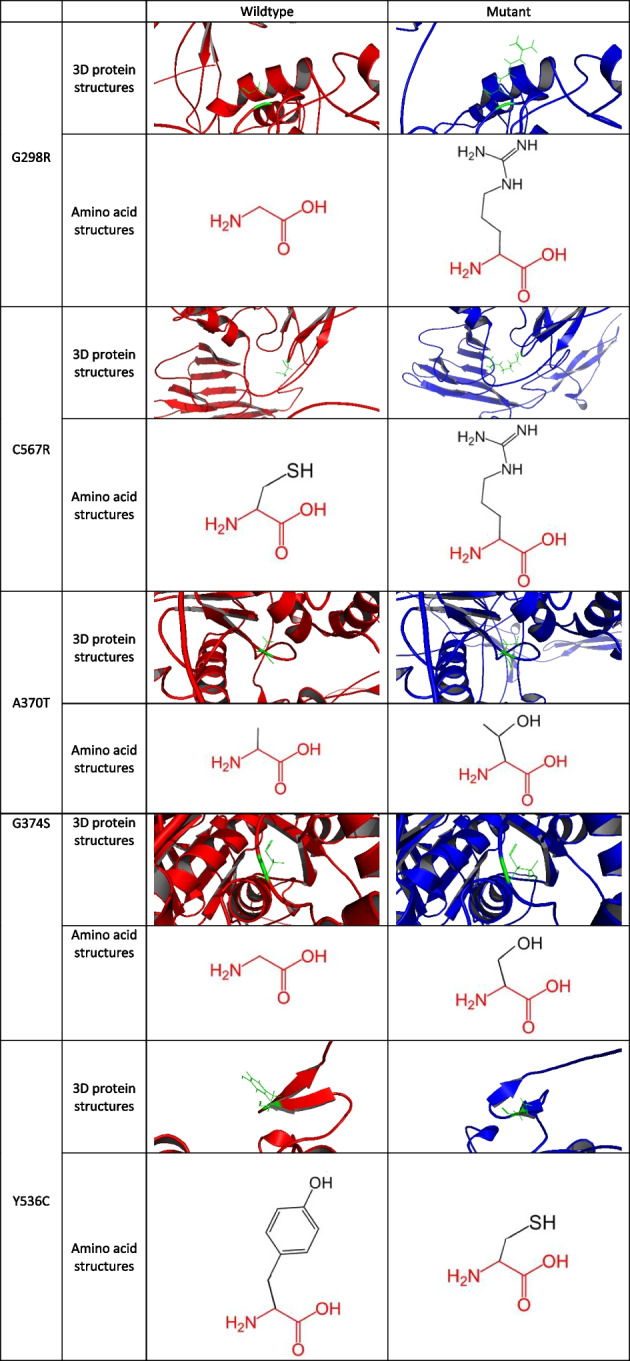
Changes in 3D protein structures and amino acid structures of important mutations (G298R, C567R, A370T, G374S (the most deleterious mutations according to Table 3) and Y536C (caused a substantial disruption in the secondary structure nearby)). The mutation sites are colored green

We study the effects of each mutation and how they affect the structures nearby. The impact of 50 pathogenic ADAMTS3 non-synonymous single nucleotide polymorphisms (nsSNPs) on amino acid sizes, charges and hydrophobicity is analyzed by Project HOPE. Among these nsSNPs, 26 caused a decrease in amino acid size, whereas 22 led to an increase. At 23 sites, the charge was altered, with 20 changing from positive to neutral, one changing from neutral to positive, and two changing from negative to neutral. Hydrophobicity was decreased in seven mutations, while 22 others resulted in an increase. These findings suggest that alterations in amino acid properties at these positions have the potential to impact protein structure and its interactions with other molecules, ultimately affecting protein function (see Additional file 7: File S7). The local 3D structures of the aforementioned 25 mutations incorporated into the AlphaFold protein models were also investigated (Fig. 5, Additional file 8: File S8). The results show that most of the mutations do not have large effects on the structure of the sequence near the mutational position in the 25 mutations. Only Y536C has a substantial disruption in the secondary structure nearby when compared with other mutations.

### Post-translational modification (PTM) prediction

We predicted common post-translational modification (PTM) including methylation, phosphorylation, ubiquitination and glycosylation for the wildtype and mutant structure. GPS-MSP predicted there are no methylated sites in ADAMTS3. The predicted phosphorylation sites at serine, threonine and tyrosine by various kinases differ between NetPhos 3.1 and GPS 6.0, with GPS 6.0 predicting more phosphorylation sites than NetPhos 3.1 for both structures (see Additional file 9: File S9). Notably, some phosphorylation sites appear and disappear after the mutation. GPS 6.0 shows disappearance of phosphorylation sites at Ser58, Tyr536, Tyr636 and Thr668 and appearance of new sites at Ile291, Ala370, Pro371, Gly374, Gly412 and Gly983, while NetPhos 3.1 shows disappearance of sites at Tyr56, Ser58 and Ser957 and appearance of new sites at Ile291, Pro371, Gly374, Gly412 and Gly983. Most of these changes are at the sites of mutations involving serine, threonine and tyrosine. It is spotted that more changes in phosphorylation sites are in segment 1. For ubiquitination, UbPred found 9 lysine residues of ubiquitination sites in wildtype and mutant structure, while BDM-PUB found 37 and 36 ubiquitinated lysine residues in wildtype and mutant protein respectively, and after mutation, there are a few new and disappearance of ubiquitination sites, more of which are in segment 3 (see Additional file 10: File S10). The analysis using NetOGlyc4.0 predicted all possible O-glycosylation sites in both proteins, and some mutants lost or gained glycosylation at specific positions, a majority of which are located in segment 3 (see Additional file 11: File S11).

### Molecular dynamics simulation

**Fig. 6:**
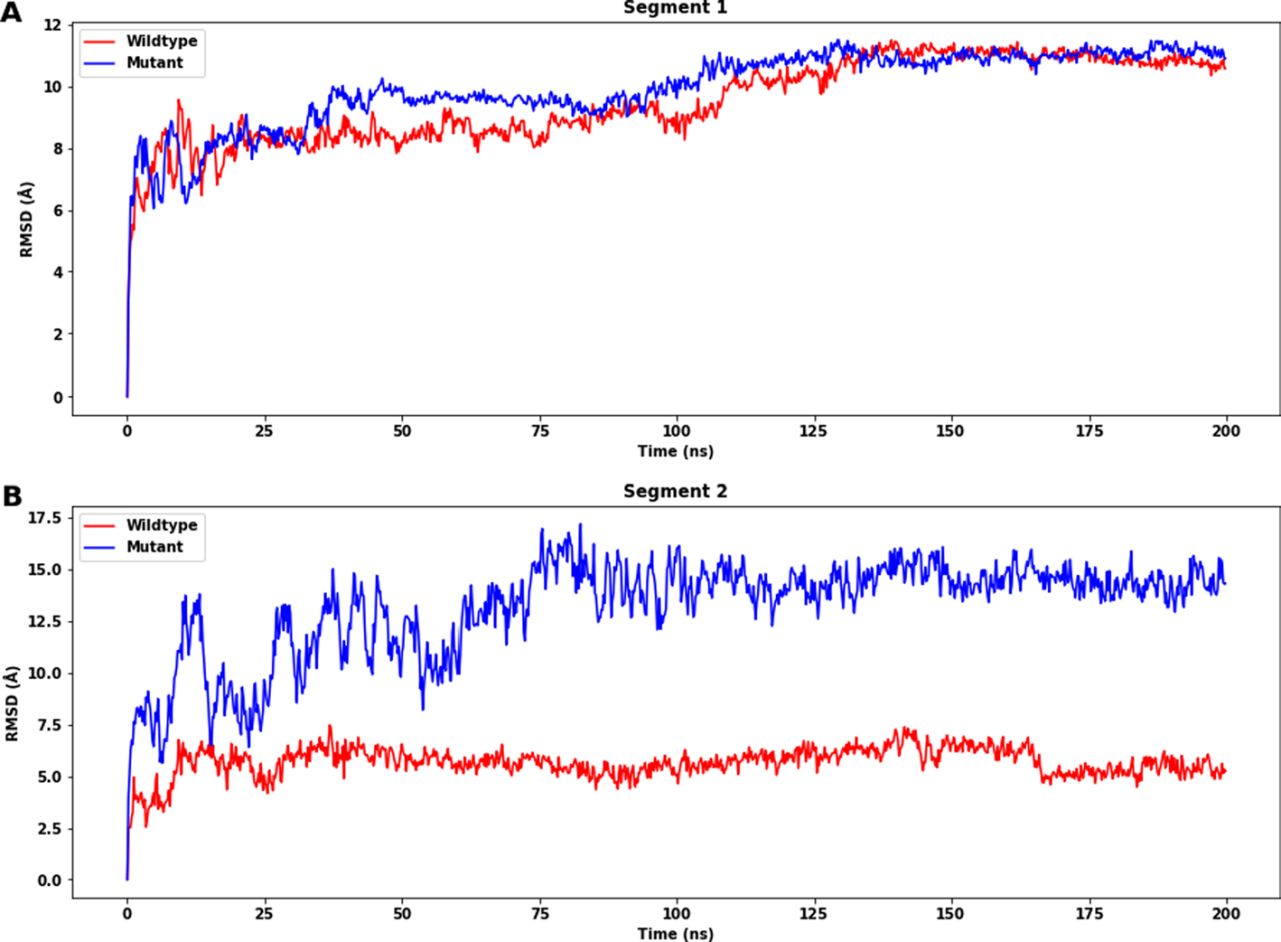
Root-mean-square deviation (RMSD) of the C-alpha atoms of wildtype (red) and mutant (blue) of segments 1 and 2 with time. **A** For segment 1, the difference in equilibrium RMSDs is insignificant (wildtype: mean: 10.830 Å, SD: 0.169 Å; mutant: mean: 11.109 Å, SD: 0.157 Å). **B** For segment 2, there is a significant difference between the RMSDs (wildtype: mean: 5.31 Å, SD: 0.344 Å; mutant: mean: 14.312 Å, SD: 0.584 Å). All the means and SD are calculated with values after 170 ns

Figure 6 illustrates the change in RMSD values over time for protein C-alpha atoms. For segment 1, the systems of both the wildtype and mutant structures reach equilibrium after 130 ns, after which the RMSD values of the two structures do not differ significantly (wildtype: mean: 10.830 Å, SD: 0.169 Å; mutant: mean: 11.109 Å, SD: 0.157 Å), which shows that the mutations at segment 1 do not affect the structure a lot. However, for segment 2, the wildtype protein reaches stability in just under 10 ns. Following that, the system equilibrated, and the simulation converged for the duration of the run, but the RMSD values of the mutant protein fluctuate more as compared to wildtype structure throughout the simulation. The mutant structure has a greater RMSD (wildtype: mean: 5.31 Å, SD: 0.344 Å; mutant: mean: 14.312 Å, SD: 0.584 Å). This indicates that the wildtype structure is more stable than the mutant one for segment 2. All the means and SD are calculated with values after 170 ns.

**Fig. 7:**
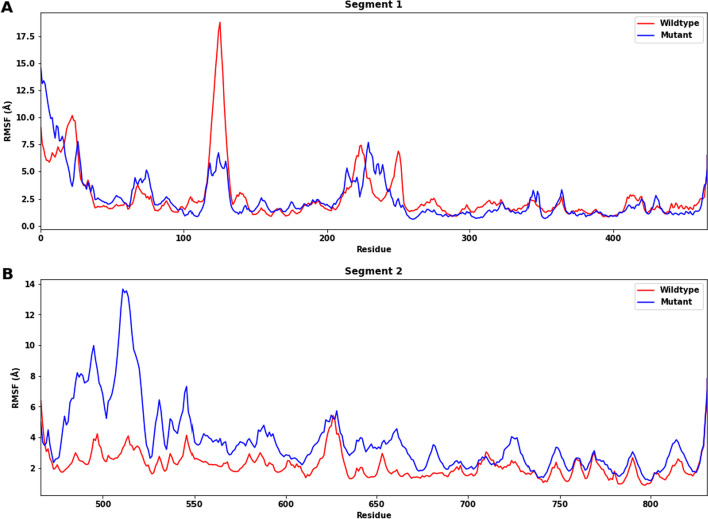
Root-Mean-Square Fluctuation (RMSF) for each residue of wildtype protein (red) and mutant protein (blue) of segments 1 (**A**) and 2 (**B**). **A** The RMSFs of the wildtype and mutant structures are similar in general. **B** The overall RMSF of the mutant structure is higher than that of the wildtype one

Regions of the proteins that fluctuate the most during the simulation are shown by peaks on the RMSF plots (Fig. 7). β-strands and α-helices are often stiffer and less variable than the protein's unstructured component. In segment 1, although the peak at residues Asn119-Pro129 is larger for the wildtype structure, the RMSFs of the wildtype and mutant structures are similar in general. This shows that the mutations in segment 1 do not stabilize or destabilize the structure significantly. In segment 2, the overall RMSF of the mutant structure is higher than the wildtype one, suggesting that the mutations destabilized the structure at that segment. There is a huge difference in RMSFs in residues Met478-Pro523, which indicates that this region is the most destabilized.

**Fig. 8:**
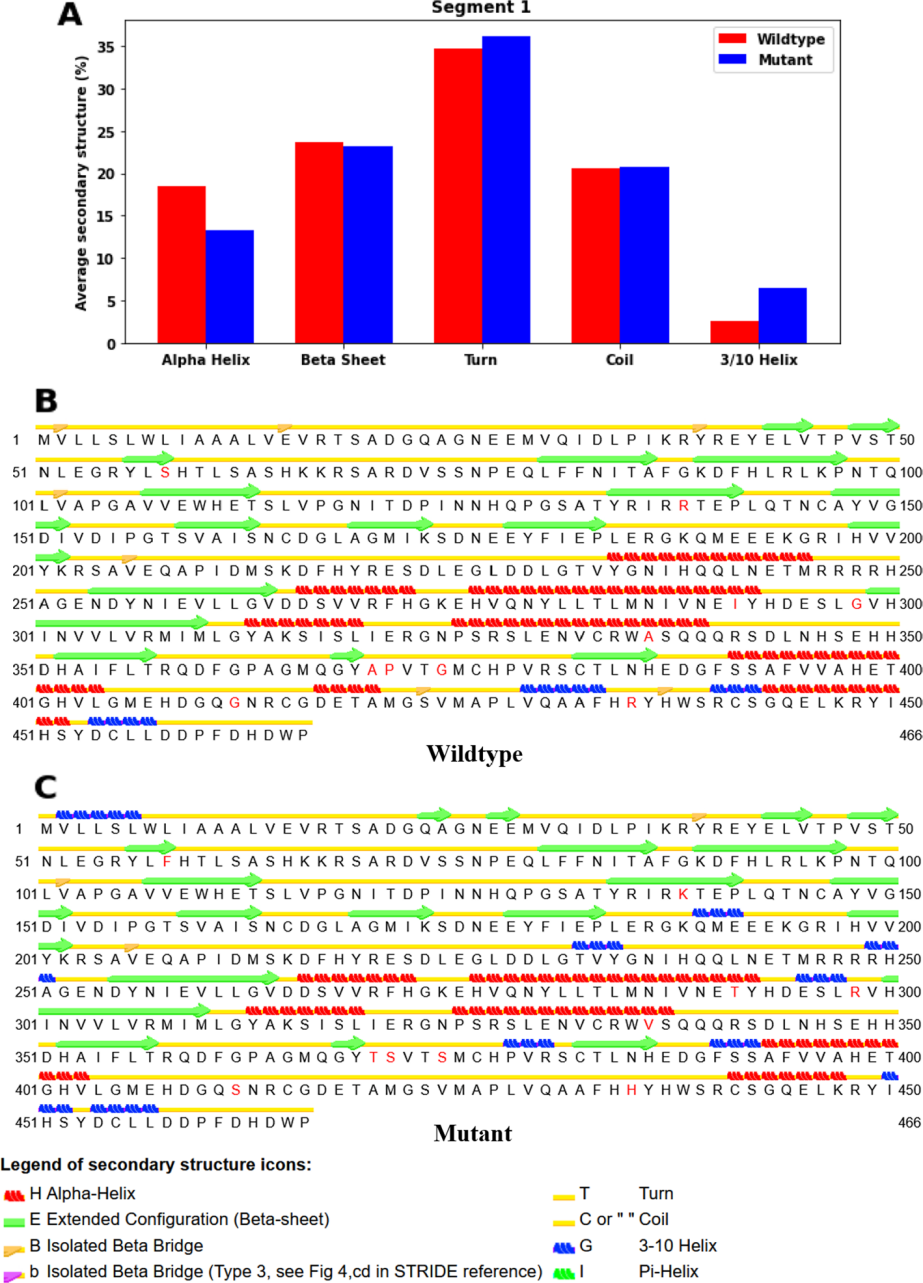
Analysis of secondary structure in segment 1 after 170 ns of MD simulation. Average secondary structure of wildtype and mutant structures (**A**). Secondary structure elements of wildtype (**B**) and mutant (**C**) structures. The mutating amino acids are marked red

**Fig. 9:**
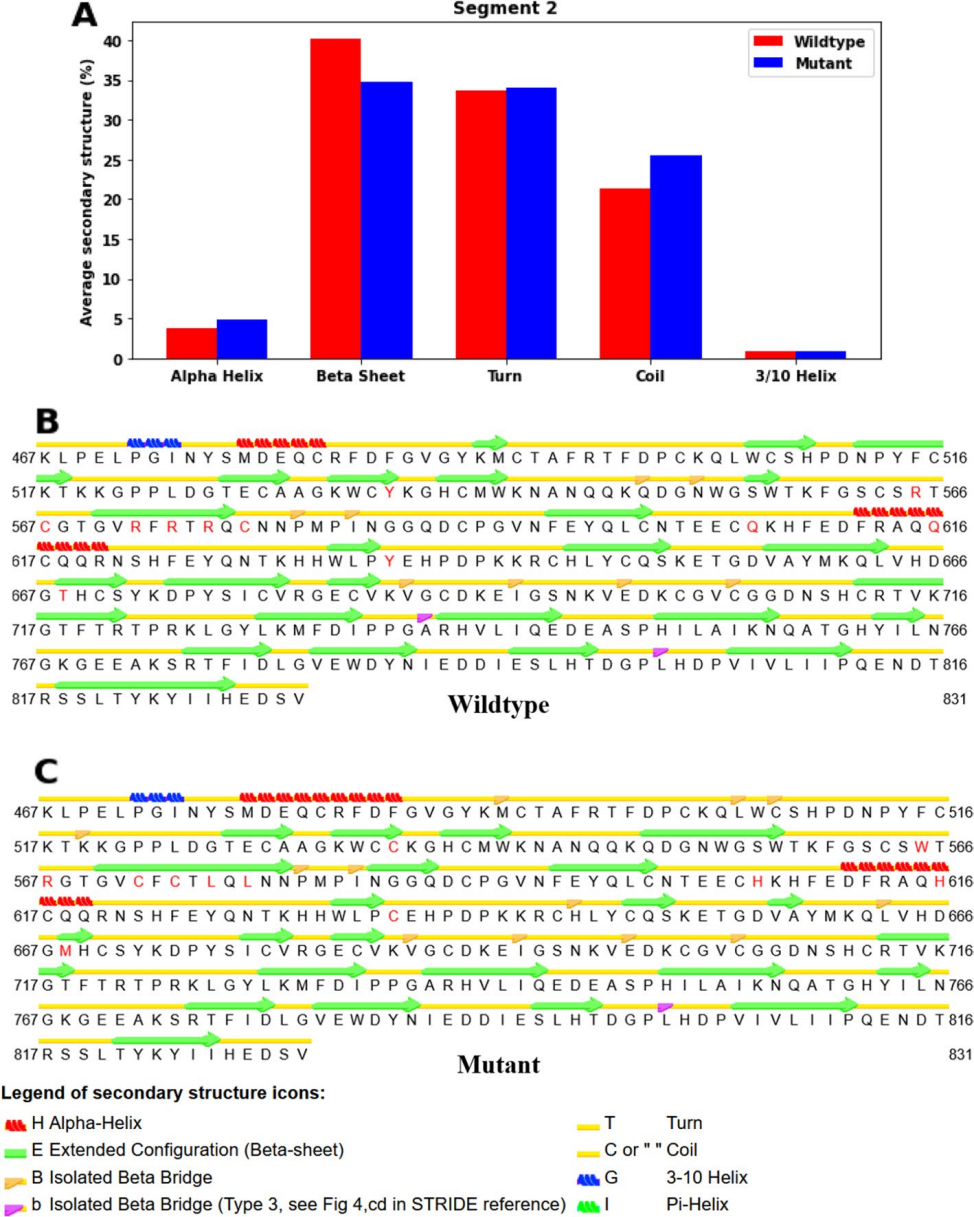
Analysis of secondary structure in segment 2 after 170 ns of MD simulation. Average secondary structure of wildtype and mutant structures (**A**). Secondary structure elements of wildtype (**B**) and mutant (**C**) structures. The mutating amino acids are marked red

The average structures during the simulation after 170 ns are also calculated, and their secondary structures are analyzed. For segment 1 (Fig. 8A–C), the percentage of the average secondary structure of α-helices has dropped by 5.15% in mutant structure when compared to the wildtype one, but there is a 3.86% increase in the percentage of 3_10_ helices, which might stabilize the mutant structure and counteract the destabilizing effects by the disruption of α-helices by the mutations, and a 1.50% increase in the percentage of turns. For segment 2 (Fig. 9A), there is a 5.48% drop in the percentage of β-sheets and a 4.11% increase in that of coils, which could destabilize the overall structure of the mutant protein in that segment. Apart from that, the percentage of α-helices has also increased by 1.10%. Looking into the distribution of the change in secondary structures in different residues in segment 2 (Fig. 9B, C), we see that there are disruptions of β-sheets in resides Lys491-Met492, Trp506-His509 and Asn512-Thr518, which might be the cause of an increase in RMSFs at resides Met478-Pro523 in the mutant structure. We also see that there are no huge changes in the secondary structures near the position of mutations of most of the SNPs. There is no SNP in the residues of the aforementioned disruptions of β-sheets.

**Fig. 10:**
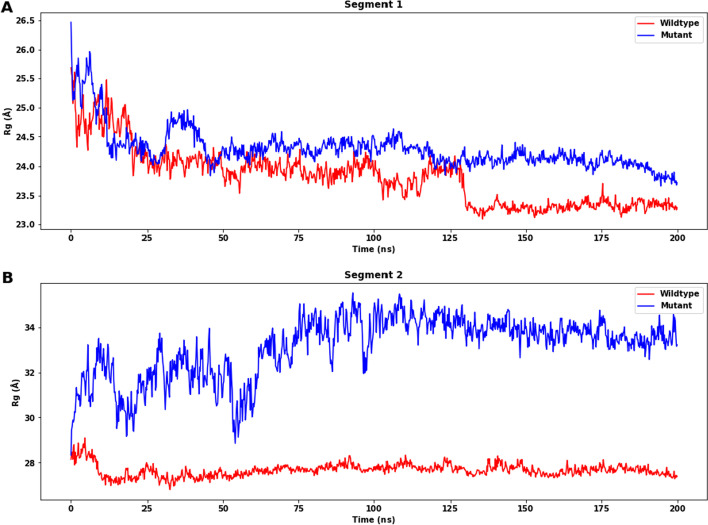
Radii of gyration (Rg) of wildtype protein and mutant protein of segments 1 and 2. **A** For segment 1, the difference in Rg is not large (wildtype: mean: 23.339 Å, SD: 0.082 Å; mutant: mean: 23.984 Å, SD: 0.139 Å). **B** For segment 2, there is a significant difference between the Rg (wildtype: mean: 27.648 Å, SD: 0.163 Å; mutant: mean: 33.564 Å, SD: 0.402 Å). All the means and SD are calculated with values after 170 ns

Analysis of the radius of gyration (Rg) is also conducted. The two most crucial indicators for determining a macromolecule’s structural activity are Rg determination and calculations of distance. The rate at which a protein folds is proportional to its compactness and can be measured using a sophisticated computer method for calculating the gyration radius. From Rg (radius of gyration) analysis of wildtype and mutant structures, it can be observed that the mutant one revealed overall higher Rg values over the simulation time scale when compared to those of wildtype in both segments 1 and 2, but the difference for segment 1 is not as significant as that for segment 2. As a result, the flexibility of the mutant is increased (Fig. 10A, B).

**Fig. 11:**
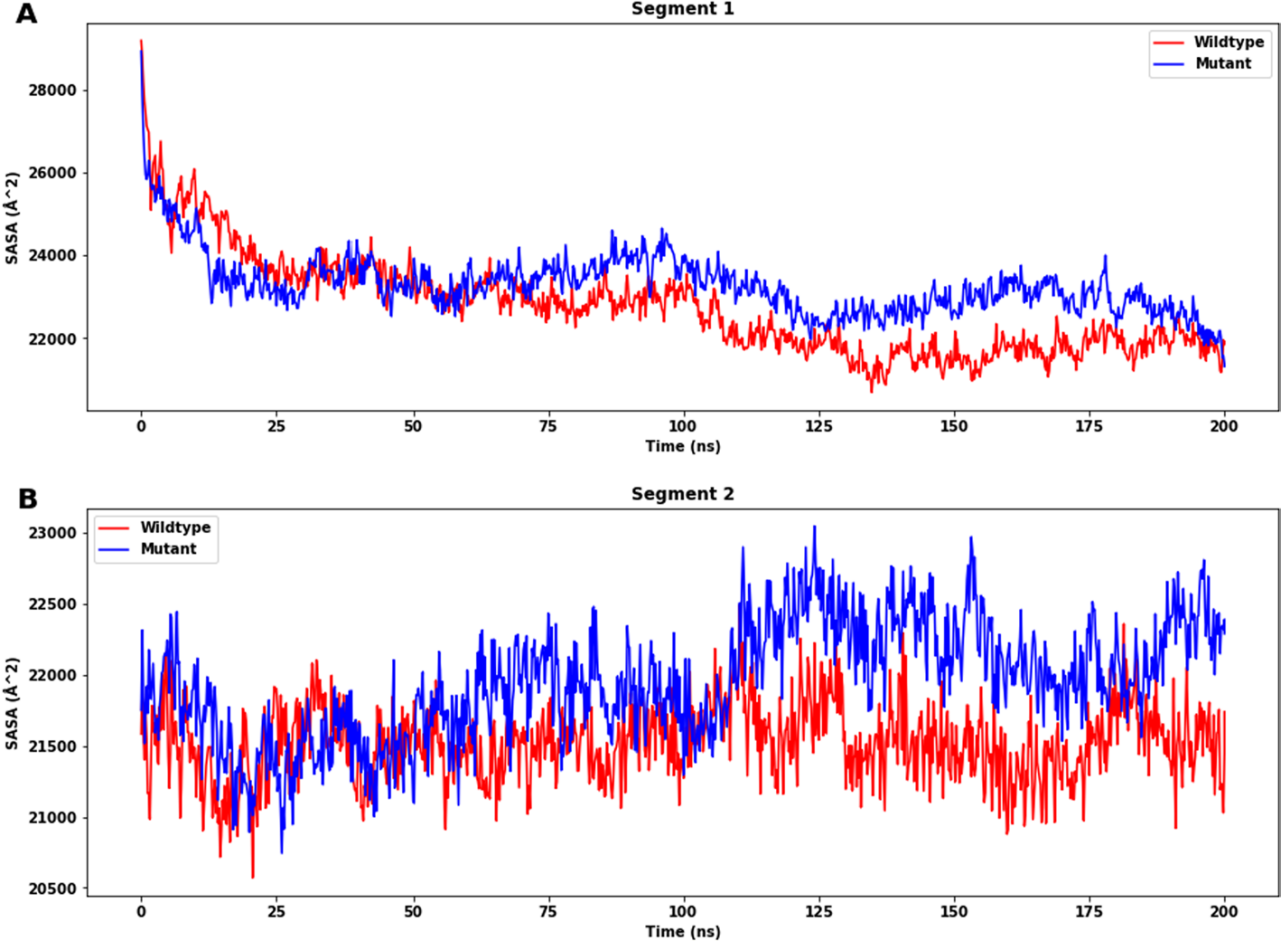
The solvent accessible surface area (SASA) (Å^2^) of wildtype and mutant protein of segments 1 (**A**) and 2 (**B**). For both segments, the SASA (Å^2^) of the mutant structure is higher than that of the wildtype one (Segment 1 wildtype: mean: 21,906.066 Å^2^, SD: 282.987 Å^2^; mutant: mean: 22,675.036 Å^2^, SD: 453.033 Å^2^; segment 2 wildtype: mean: 21,565.973 Å^2^, SD: 245.14 Å^2^; mutant: mean: 22,160.942 Å^2^, SD: 269.095 Å^2^). All the means and SD are calculated with values after 170 ns

Solvent accessible surface area (SASA) analysis indicated that the mutant structure has a higher SASA value than wildtype for both segments 1 and 2 (Fig. 11A, B). Since a higher SASA value denotes protein expansion, it can be suggested that wildtype is more stable than the mutant protein. The reason for a greater change observed in the SASA value could be the effect of amino acid substitution by altering the size of the protein surface and other characteristics.

**Fig. 12:**
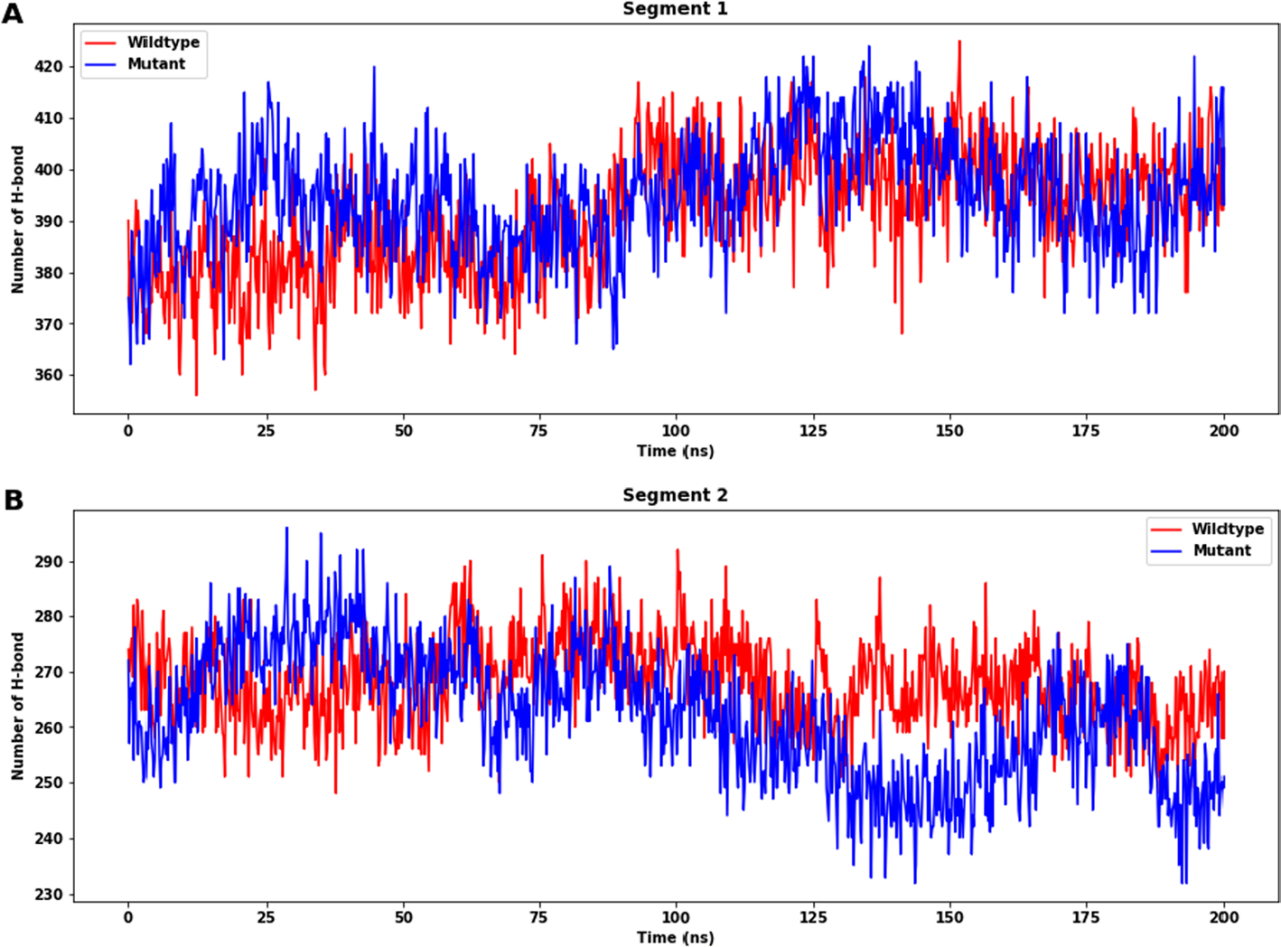
The total number of H-bond count throughout the simulation of wildtype and mutant protein of segments 1 and 2. **A** For segment 1, the difference in the number of H-bonds for is not large (wildtype: mean: 396.854, SD: 7.427; mutant: mean: 392.351, SD: 9.540). **B** For segment 2, there is a larger difference between the SASA (wildtype: mean: 263.192, SD: 6.421; mutant: mean: 255.709, SD: 9.473). All the means and SD are calculated with values after 170 ns

The total number of H-bonds within the proteins was also calculated during the MD simulation as depicted in Fig. 12A, B. The difference between the number of H-bonds for segment 1 is insignificant, again indicating that the effect of destabilization is small for mutations in this segment. For segment 2, it can be noted that the wildtype structure forms a greater number of H-bonds while the mutant one exhibit a fewer number of H-bonds, which may affect the stability of the mutant protein.

### Principal component analysis (PCA)

In this study, PCA was used to analyze the trajectories and structures of the wildtype and mutant proteins in segments 1 and 2. The plots generated from PCA analysis show the collective motions of the system projected onto the first two principal components.

**Fig. 13:**
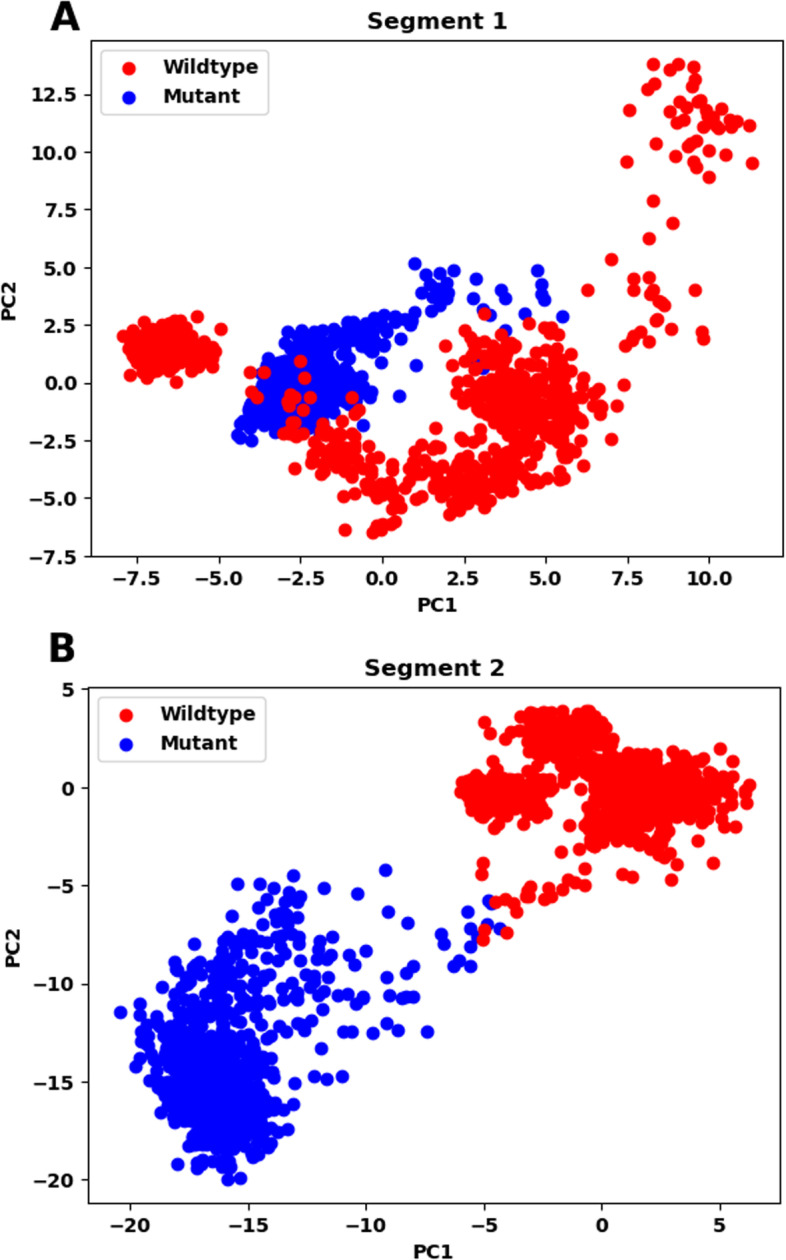
The PCA plots for trajectory analysis of segments 1 (**A**) and segment 2 (**B**). The wildtype and mutant trajectory data are represented by blue and red dots respectively

The plots of segments 1 and 2 (Fig. 13A, B) indicate that there is a significant difference in the trajectories and motions of the wildtype and mutant protein systems. In segment 1, the plots of the wildtype and mutant structures overlap to a great extent, indicating that the mutations in this segment have little effect on the collective motions of the protein. However, in segment 2, there is less overlap between the plots of the wildtype and mutant structures, suggesting that the mutations in this segment have a greater impact on the collective motions of the protein.

### Free energy landscape (FEL)

**Fig. 14:**
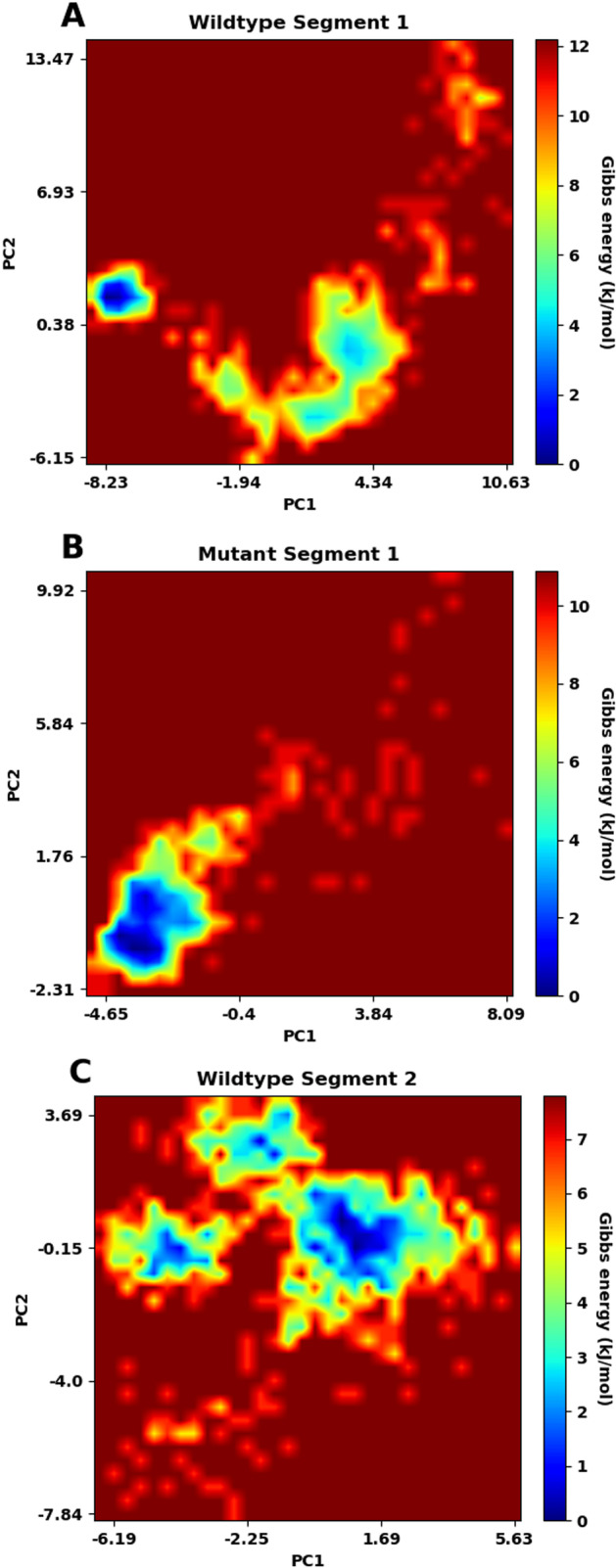

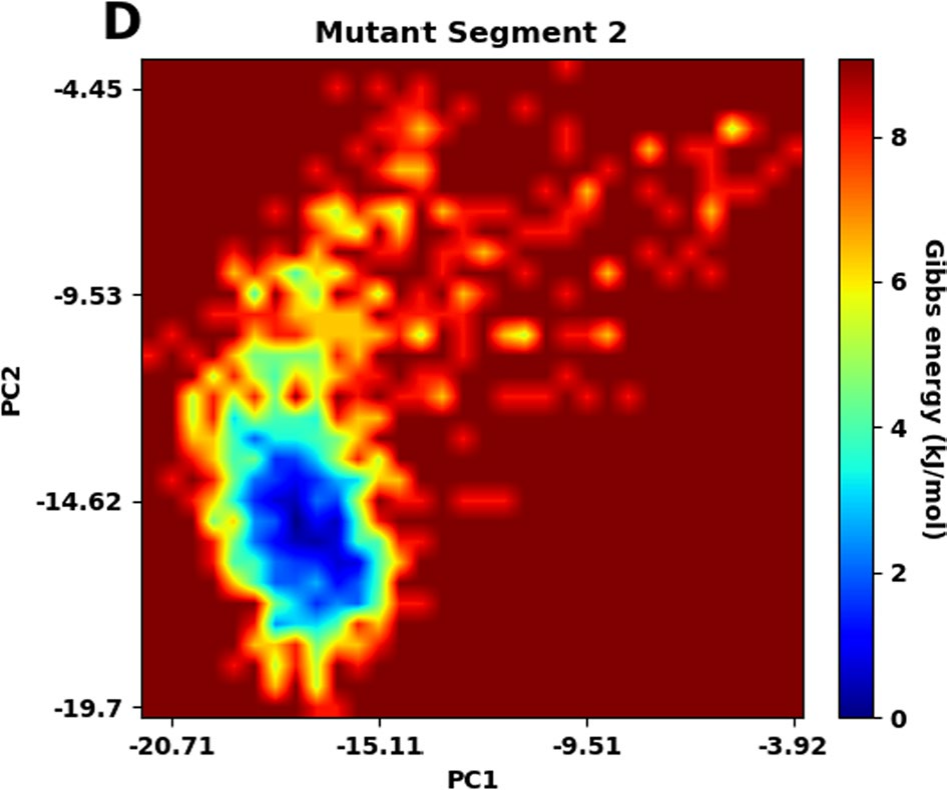
Free energy landscape (FEP) analysis. Gibbs energy is plotted as a function of the first 2 principal components (PC1 and PC2) for wildtype segment 1 (lowest energy = 12.2 kJ/mol) (**A**), mutant segment 1 (lowest energy = 10.9 kJ/mol) (**B**), wildtype segment 2 (lowest energy = 7.80 kJ/mol) (**C**) and mutant segment 2 (lowest energy = 9.08 kJ/mol) (**D**). The conformation with the lowest energy is indicated by a deep blue color

FEL plots are made after the PCA analysis using the first two principal components. In the plots of FEL (Fig. 14A–D), the conformation with the lowest energy is indicated by a deep blue color. For segment 1, the lowest energy for the wildtype structure is 12.2 kJ/mol, while that for the mutant one is 10.9 kJ/mol. For segment 2, the lowest energy for the wildtype structure is 7.80 kJ/mol, but that for the mutant structure is 9.08 kJ/mol. For both segments, the wildtype and mutant structures show differences in the numbers and the positions of stable conformations, which correspond to the local minima in the FEL plots. These suggest that the mutations have affected the overall conformational stability of the protein.

## Discussion

The lymphatic system plays a crucial role in maintaining the fluid and tissue balance of the body. The transcription factors PROX1 and SOX18n collaborate to induce the growth and differentiation of lymphatic endothelial cells from veins. Primary lymphedema, which can be solitary or syndromic, may result from the malfunction of several other proteins that are essential for lymphatic system development. To date, 27 genes have been associated with primary lymphedema [24]. The present study investigated the impact of SNPs on the structure and function of the ADAMTS3 protein using cutting-edge *in-silico* techniques. Specifically, 848 nsSNPs of the ADAMTS3 gene were identified in the dbSNP database, 919 in the Ensembl database, and only 16 of the known MAFs of nsSNPs in the ADAMTS3 gene were less than 1%. SIFT and PolyPhen-2 confirmed that 50 nsSNPs in the ADAMTS3 gene are high-risk and deleterious.

The top five nsSNPs with high risk are highly deleterious, as indicated by all cutting-edge prediction techniques utilized in this study (Additional file 1: File S1). These five nsSNPs (G298R, C567Y, A370T, C567R, and G374S) could be used as a marker for other patients with mutations in the ADAMT3 gene, even if they have not yet been associated with Hennekam syndrome caused by ADAMTS3. Three of these nsSNPs (A370T, C567Y, and C567R) were found to disrupt the function and structure of the ADAMTS3 protein with MUpro and INPS3D. These SNPs have the potential to contribute to the pathophysiology of ADAMTS3-related disorders, such as Hennekam syndrome. Some of the nsSNPs that we have described have been shown to be pathogenic in patients with Hennekam syndrome due to mutations in the ADAMTS3 gene [5].

In the current study, we utilized the same in silico procedures as described previously for different genes to determine lower numbers of nsSNPs [25, 26]. Like our study, we predicted missense mutations for the ADAMTS3 gene that are deleterious and have been demonstrated to destabilize the protein's structure and function. Similar studies have been conducted using the same protocols to predict nsSNPs for various genes and disorders [26]. Computational analysis of nsSNPs of the ADA gene in Severe Combined Immunodeficiency, through similar protocols, showed one mutation, while studies on TCGR1 and CCBE1 showed more than 10 mutations [26–28].

In selecting highly pathogenic variations, we considered the effects of non-synonymous single nucleotide polymorphisms (nsSNPs) on sequence conservation, sequence features, and structural factors [29]. We used a comprehensive selection of cutting-edge techniques to predict pathogenic nsSNPs based on wide-ranging approaches [29]. We confirmed the predictions of SIFT and PolyPhen-2 using 16 other tools, including PANTHER-PSEP, PhD-SNP, PROVEAN, SNPs&GO, SNAP2, PON-P2, VarCards (FATHMM, CADD, LRT, M-CAP, MutationTaster, Mutation Assessor, MetaLR, FATHMM MKL Coding), PMut, and MutPred2. These computational techniques present different prediction characteristics based on various databases. According to a previous study based on ClinVar benchmark results, PolyPhen-2, MutationTaster, and SIFT have elevated sensitivity [30]. For instance, a study employing benchmark data from ClinVar, TP53, and PPARG found values between 65.00 and 76.99%. Despite PROVEAN's high specificity, Mutation Taster, PolyPhen-2, and SIFT have higher sensitivity than other tools [30]. Therefore, we included VARCARDS, SIFT, PROVEAN, Mutation Taster 2, and PolyPhen-2 in our prediction analysis. We also used databases such as SNAP2, PON-P2, MutPred2, PhD-SNP, PANTHER-PSEP, and PMut to speed up the polymorphism calculation.

Numerous biological procedures and processes depend on amino acids, especially highly modified and conserved protein interactions. SNPs in conserved loci can cause more significant deleterious effects than those in non-conserved loci [31]. In our study, we detected a total of 26 nsSNPs, of which 19 (Q927R, G298R, C567Y, C567R, Q616H, R565W, R565Q, P371S, P513T, R248H, T668M, R435H, N98S, R883C, G412S, L801F, S1038F, G983S, R959W) were found at evolutionarily conserved regions and important functional residues. The remaining nsSNPs, which were located on either exposed or buried residues, were not expected to have any significant structural or functional importance in the ADAMTS3 protein. Although these 19 ADAMTS3 nsSNPs have not yet been identified in individuals with Hennekam syndrome, they would be considered pathogenic nsSNPs if they were found in the future.

Protein stability is a critical factor for the biological function, activity, and control of biomolecules. Pathogenic missense mutations often result from improper folding and reduced stability of proteins [32, 33]. Protein thermodynamic stability is determined by measuring the folding free energy (ΔG), which is the energy difference between the folded and unfolded states. Generally, a ΔΔG > 0 indicates enhanced stability, while a ΔΔG < 0 indicates decreased stability in the mutant protein. MUpro and INPS3D predicted that 42 of the 50 high-risk nsSNPs would impair protein stability, while the remaining 8 nsSNPs had negative ΔΔG values according to at least one of the two approaches. However, when assessing mutations based on ΔΔG other than zero, it is crucial to consider that a mutation may lead to significant structural changes in the protein, depending on the relative values of ΔG and ΔΔG [31]. Proteins undergo conformational changes to become functional [34]. COACH and RaptorX Binding ligand-binding site servers predicted that the high-risk nsSNPs G365, M366, Q367, G368, Y369, V395, H398, E399, H402, H408, A426, P427, L428, and V429 are ligand-binding sites. ConSurf assigned them a score of 9, indicating that they are highly conserved and could be used to monitor harmful mutations. SNPs are more common in conserved positions [35]. Among them, H398, H402, and H408 were predicted by COACH and RaptorX based on higher pocket scores, to serve as a binding site for drug designing purposes [36]. The previously described mutations I291T and I168P, together with all the newly predicted missense mutations, destabilized the protein activity [5]. To examine the biophysical characteristics of these variations, the Project HOPE tool was utilized, revealing some differences in the properties of the amino acids in the mutations, which could lead to disruption of the structure and hence misfolding. Additionally, the results from Project HOPE showed that many mutations did not affect their neighboring secondary structures in the sequences, but they were still deleterious, suggesting that they affected structures far away from the mutation positions. For example, the formation of tertiary structures might cause interactions between the mutation position and another region, resulting in a remote effect. Therefore, changing an amino acid at a specific site might disrupt the protein structure and induce misfolding.

Experiments are considered more reliable in classifying pathogenic mutations. However, conducting recurrent experiments on all non-synonymous single nucleotide polymorphisms (nsSNPs) is time-consuming. Therefore, the methods used in this analysis support the various outcomes of SNPs that illustrate pathogenicity. The importance of deleterious nsSNPs that affect a potential phosphorylation site was highlighted by post-translational modification (PTM) prediction. Modeling of the wildtype and mutant proteins is a reasonable assumption, but it should be noted that even small changes in one segment can potentially affect the overall stability and conformation of the protein, ultimately affecting its function. Hence, it is crucial to analyze each segment separately to understand the potential impact of the mutations. PTM prediction is relevant in the context of nsSNPs as PTMs can be affected by changes in the amino acid sequence resulting from nsSNPs. PTMs are chemical modifications that occur after protein translation and can alter protein function, localization, stability, and interactions with other molecules. If an nsSNP occurs within a PTM site, it may affect the likelihood or efficiency of PTM occurrence, which can in turn impact protein function. For instance, if an nsSNP causes a change in the amino acid sequence at a phosphorylation site, it may alter the ability of kinases to recognize and phosphorylate the site, affecting downstream signaling pathways. PTM prediction tools can help identify potential PTM sites within a protein sequence and assess the impact of nsSNPs on these sites. This information can be useful in understanding the potential functional consequences of nsSNPs and prioritizing nsSNPs for further investigation.

The MD simulation studies were introduced to explore the dynamic behavior and stability of the protein. The RMSD, RMSF, secondary structure, Rg, SASA and hydrogen bonding analyses suggest that the mutations have different effects on the stability of the two segments of the ADAMTS3 protein. After including the 25 mutations, segment 2 shows a more significant destabilization effect than segment 1. The analysis of secondary structure shows that in segment 1, the mutations do not significantly affect the stability of the structure, but they may alter the distribution of secondary structures. In segment 2, the mutations lead to destabilization of the structure, particularly in the region of residues Met478-Pro523, which show higher RMSFs and disruption of β-sheets. This secondary structure analysis also shows the remote effect in Project HOPE analysis—the secondary structures next to most of the mutation sites are not disrupted, but the disturbances occur at another part within the segment, again suggesting that the effects of mutations act via tertiary interactions. Additionally, the mutant structure exhibits a higher RMSD, RMSF, Rg and SASA compared to the wildtype for segment 2, indicating greater flexibility and suggesting that the mutations may affect the overall compactness and shape of the protein. The decrease in the number of H-bonds in the mutant structure compared to the wildtype further supports the destabilizing effect of the mutations, which aligns with the fact that β-strands are disrupted in segment 2. These findings could provide insights into the functional consequences of the mutations and guide further experimental investigations. However, the effect of the mutations on segment 1 is relatively small, with little difference in RMSD, RMSF, Rg and the number of H-bonds observed between the wildtype and mutant structures, which might be explained by the fact that the mutations increased the percentage of α-helices when there is a decrease in β-strand percentage. This suggests that the main stabilization effects of the mutations come from segment 2. Since we did not include C567Y in our protein model as it is at the same position as another mutation C567R, the effects of C567Y have not been extensively studied in MD simulation. Further studies are needed to show how it affects the protein.

It is noted that among the 25 mutations included in modeling the mutant structure, 4 of them, namely I291T, A370T, Y536C and C567R, are predicted to affect the stability of the protein by MUpro and INPS3D. 2 of them (I291T and A370T) are in segment 1 while the other 2 (Y536C and C567R) are in segment 2. Despite the 2 destabilizing mutations in segment 1, it is found with MD simulation that segment 1 does not have significant destabilization effects when they exist in the model. Since MUpro and INPS3D predict the effects of each mutation individually without considering other mutations, while MD simulation uses a mutant model that incorporates multiple mutations at the same time to study their effects, this suggests that the combined effects of multiple mutations are not just a simple superposition of individual mutations, which is indicated by the insignificant destabilization in segment 1 although the 2 destabilizing mutations (I291T and A370T) are included. When both destabilizing mutations, together with other mutations, exist, the destabilizing effects were alleviated. This might be evidence that compensatory effects of mutations are present in ADAMTS3. Similar intragenic compensatory mutations were also observed in various organisms and viruses such as DNA Bacteriophage φX174 [37] and influenza A virus [38]. It is worth investigating the mechanism of the compensatory effects of ADAMTS3 and whether they share a common mechanism in different biological systems.

The fact that the stability of segment 1 is not destabilized, but the mutations are still deleterious might be rationalized by the results in the PTM analysis. We showed that there are a lot of changes in phosphorylation sites in segment 1. Since phosphorylation is a crucial mechanism for the activation and deactivation of enzymes in organisms [39], the mutations might destroy some phosphorylation sites, making the protein unable to activate or deactivate, which affects its function substantially. This provides a possible mechanism explaining why the mutations are deleterious without destabilizing the protein to a great extent.

Principal component analysis indicates a difference in the trajectories of the two segments after incorporating the mutations. FEL plots of the two segments of the wildtype and mutant structures further show that the stable conformations and Gibbs free energy change are not the same, which might change the shape in its stable form, preventing the protein from working properly.

In silico studies also have limitations, the first of which is that the reported causative nsSNPs were only partial, necessitating laboratory confirmation to confirm the findings. Our results provide data on the ADAMTS3 gene's pathogenic nsSNPs, protein 3D structure, potential PTMs sites, and gene–gene interaction, which could aid future research into the gene's function in Hennekam syndrome and other related disorders.

## Conclusion

We identified 22 deleterious mutations, 5 of which (G298R, C567Y, A370T, C567R and G374S) are exceptionally deleterious. We also included 4 other mutations previously discovered clinically. They have various effects on post-translational modification and ligand binding. Some of them were also shown to destabilize ADAMTS3. Further molecular dynamics simulation by modeling with 21 deleterious mutations and 4 clinically discovered mutations suggests that these mutations have various effects on different parts of the protein. These studies can help future research on the important SNPs for causing the disease. The association between the predicted deleterious SNPs and HKLLS3 can be tested by in vivo studies in animal models and clinical studies in a large population.

## Methods

### Collection of ADAMTS3 gene data

The Human SNP information for the ADAMTS3 gene was compiled using multiple online sources, including Mendelian Inheritance in Man (OMIM) [40], NCBI dbSNP (https://www.ncbi.nlm.nih.gov/snp). Specifically, missense SNPs were collected by selecting them from the list and downloading the resulting list of missense SNPs in the ADAMTS3 gene by clicking “Send to” and then “File”. From this list, only the Reference SNP cluster ID [41] was used. Additionally, information was gathered from the UniProt database [42]. The assembly process was conducted with adherence to scientific standards.

### Prediction through SIFT and PolyPhen-2

The nature of non-synonymous single nucleotide polymorphisms (nsSNPs), whether they are deleterious/damaging or tolerated, was determined in this study using two methodologies, namely SIFT and PolyPhen-2. SIFT predicts harmful nsSNPs by analyzing protein homology sequence and natural nsSNP alignments. When the score is less than 0.05, SIFT considers the effect of nsSNPs on protein function to be deleterious [43]. On the other hand, PolyPhen-2 assesses the structural and functional consequences of a protein by analyzing its amino acid sequence and substitutions. PolyPhen-2 classifies a mutation or substituted amino acid in a query protein as “Probably Damaging” (score > 0.98), “Possibly Damaging” (0.446 < score ≤ 0.908), or “Benign” (score ≤ 0.446) [44].

### Missense mutations’ functional effects evaluation

The results obtained from the SIFT and PolyPhen-2 methodologies were further validated by other computational algorithms, including Mutation Assessor, PON-P2, SNPs&GO, PROVEAN, PMut, SNAP2, PANTHER, VarCards, and PhD-SNP, to confirm the functional consequences of missense mutations. The PROVEAN algorithm was used to calculate the detrimental effects of nsSNPs on ADAMTS3 protein sequences. In cases of homologous sequence, delta alignment scores were used by these tools to compare protein sequence and variant versions. An alignment score of ≤ -2.5 indicated a harmful nsSNP [45]. The SNAP2 algorithm, which is a neural network-based classifier, was used to predict the consequences of single amino acid alterations in ADAMTS3 protein. The server accepted a FASTA sequence and outputted a prediction score ranging from + 100 (strong effect prediction) to 100 (strong neutral) indicating the probability that a mutation would impair the native protein's ability to perform its intended function [46]. The PON-P2 algorithm was used to determine the pathogenicity of amino acid substitutions by considering the characteristics of amino acids, GO annotations, evolutionarily conserved sequences, and functional annotations based on a machine-learning-based approach. The PON-P2 classification system divides all forms into three types: pathogenic, neutral, and unknown [47].

PMut is a neural network-based algorithm that accurately predicts whether a single amino acid point mutation will be pathogenic with an 80% success rate in humans. The PMut website can distinguish between neutral variations and disease-associated protein sequences when a FASTA sequence is uploaded. A score greater than 0.5 indicates the presence of pathogenic nsSNPs [48]. SNPs&GO and PhD-SNP are important machine-learning methods that rely on the comparative conservation scores of numerous sequence alignments [49]. The PhD-SNP tool contains 36,000 detrimental and benign SNVs and generates an accuracy index score that categorizes the effect of an SNP as either disease or neutral. It was developed and tested using the ClinVar dataset [22]. PANTHER-PSEP (PANTHER-position-specific evolutionary preservation, http://pantherdb.org/apparatuses/csnpScoreForm.jsp) uses homologous proteins to identify possible ancestral protein sequences at nodes of phylogenetic trees using an evolutionary preservation measure [50]. The origins of each amino acid can be traced, and an estimate of how long it has been in its pre-existing forms can be made. The PSEP score is divided into three groups based on preservation time: “probably damaging” (preservation time > 450 my), “possibly damaging” (preservation time 450my > time > 200my), and “probably benign” (preservation time < 200my). VarCards, which gives results from the following tools: M-CAP, MetaLR, MetaSVM, FATHMM MKL Coding, Mutation Assessor, CADD, and DANN, was also used. Additionally, VarCards makes it easier for researchers, scientists, and geneticists to obtain information from a variety of sources [51].

### MutPred2’s prediction of disease-related phenotypes and amino acid substitution

In order to predict the molecular mechanism underlying a disease caused by an amino acid substitution in a mutated protein, the MutPred web server (http://mutpred.mutdb.org/) utilizes multiple aspects of protein structure, function, and evolution [23]. To predict certain structural abnormalities, PSI-BLAST, SIFT, and Pfam profiles are employed. The combined scores of these servers yield a prediction with a higher degree of accuracy.

### GeneMANIA to understand ADAMTS3 interactions with other genes

In order to investigate the association between the ADAMTS3 gene and other genes based on pathways, expression, localization, genetics, and protein interaction, GeneMANIA (https://genemania.org/) was employed [52].

To confirm the relationship between the ADAMTS3 gene and other genes based on pathways, expression, localization, genetics and protein interactions, GeneMANIA (https://genemania.org/) was utilized [52].

### nsSNPs effects on protein stability

Furthermore, the effect of nsSNPs on the stability of ADAMTS3 protein was evaluated using MUpro and INPS3D.

### INPS3D

INPS3D, a newly developed tool, is capable of predicting protein stabilities in wildtype and mutant. The INPS-MD (Impact of Non-synonymous mutations on Protein Stability—Multi Dimension) web server (https://inpsmd.biocomp.unibo.it/inpsSuite/default/index3D) was used for this purpose. This tool takes into account various aspects, such as hydrophobicities and molecular weights of the original and mutated amino acids, the difference in alignment scores, the mutation probability of the original residue, the relative solvent accessibility (RSA) of the native amino acid, and the local energy difference between the wildtype and mutant protein structures. To predict the stability changes caused by each mutation, the PDB file of the AlphaFold-predicted structure of ADAMTS3 and the mutations predicted by SIFT and PolyPhen-2 were input into INPS3D. The change in stability caused by each mutation was measured by ΔΔG [53].

### MUpro

The MUpro tool, available at http://mupro.proteomics.ics.uci.edu/, employs machine learning techniques such as vector machines and neural networks to estimate the impact of single-site amino acid substitutions on protein stability [54]. These algorithms were developed using a large dataset of mutations, allowing predictions of stability changes even in the absence of tertiary structures. The predictive power of the tool was evaluated using a dataset of 1615 mutations and a 20-fold cross-validation approach, resulting in a reliability of 84.2% when using sequence data as input.

### Predictions of ligand binding sites

#### RaptorX

RaptorX Binding (http://raptorx.uchicago.edu/BindingSite/) is a computational tool that predicts protein binding sites based on a 3D model and other features including the secondary and tertiary structure of the protein, disordered regions, solvent accessibility, and others [55]. In addition to using the uGDT (Global Distance Test) and uSeqID (Sequence Identity) criteria, the tool incorporates a *p* value and a pocket multiplicity factor when predicting binding sites. A higher score indicates a higher degree of reliability for the predicted binding pocket, specifically when the score exceeds 40.

#### COACH

To predict protein–ligand binding sites, the meta-server program COACH (http://zhanglab.ccmb.med.umich.edu/COACH/) utilizes two comparison approaches, TM-SITE and S-SITE, to recognize ligand binding templates from the BioLiP protein function database, as well as binding-specific sub-structure and sequence feature correlations. The COACH server ranks the top ten models based on cluster size, PDB hits, ligand names, consensus binding residues, and readily available, downloadable complex structures, assigning a C-Score to each model. The C-score is a measure of the predicted reliability and ranges from 0–1, with higher scores indicating greater reliability [56].

#### Conservation analysis (ConSurf)

To determine the evolutionary conservation pattern of amino acids within the protein sequence, Bayesian empirical inference was employed. This approach provides conservation scores and color schemes, where a variable amino acid is assigned a score of one and the most conserved amino acid is assigned a score of nine. The FASTA sequence of ADAMTS3 protein was submitted for ConSurf analysis [56].

#### Prediction of PTMs sites

In order to predict protein activity based on post-translational modifications (PTMs), we employed various tools to predict potential PTM sites in ADAMTS3 protein. To predict methylation sites, we used GPS-MSP 1.0 (http://msp.biocuckoo.org/online.php) [57]. For the prediction of potential phosphorylation sites, we employed GPS 6.0 (https://gps.biocuckoo.cn/online.php) and NetPhos 3.1 (https://services.healthtech.dtu.dk/services/NetPhos-3.1/) [58, 59]. NetPhos 3.1 utilizes an ensemble of neural networks to predict phosphorylation sites and a NetPhos score > 0.5 indicates phosphorylation. A higher GPS 6.0 score indicates a higher probability of phosphorylation. To determine possible methylation and ubiquitylation sites, we used GPS-MSP 1.0, UbPred (http://www.ubpred.org), and BDM-PUB (http://bdmpub.biocuckoo.org/) [17, 50]. To predict glycosylation sites, we employed NetOGlyc 4.0 (https://services.healthtech.dtu.dk/services/NetOGlyc-4.0/), which predicts glycosylation sites based on a threshold value of 0.5 for the predicted glycosylation score [60, 61].

#### Protein modeling

An accurate tool for predicting protein structure, AlphaFold 2 [62], was utilized. Both the wildtype and mutant model were constructed by the offline version of Alphafold 2 Colab [63]. The wildtype and mutant models were validated through the Ramachandran plot [64] and MolProbity all-atom contact analysis [65]. MolProbity is used to check the existence of steric clashes in order to validate the structures. It uses clashscore to measure the amount of steric clashes a protein structure has. The overlap of each pair of nonbonded atoms is calculated according to their van der Waals surfaces. If the overlap is more than 0.4 Å, that pair of nonbonded atoms are considered to have a serious clash. The clashscore is the number of serious clashes per 1000 atoms in the protein.

#### Project HOPE

In this study, we utilized a web-based program called Project HOPE to assess the impact of amino acid mutations on their physical properties, including size, charge, and hydrophobicity [66]. The structural changes resulting from these mutations were visualized using both the wildtype and mutant models generated by AlphaFold 2.

#### Molecular dynamics simulation

For the study, molecular dynamics simulations were carried out for 200 ns using the Desmond algorithm from Schrodinger LLC [66, 67]. The wildtype and mutant proteins were preprocessed with the Protein Preparation Wizard of Maestro, which included complex optimization and minimization. The System Builder software was used to prepare each system with the TIP3P solvent model and an orthorhombic box. The box dimensions were specified as a buffer distance of a = 20.0 Å, b = 20.0 Å, c = 20.0 Å. To simulate physiological conditions, the system was neutralized with counterions and 0.15 M sodium chloride (NaCl) was added. The OPLS 2005 force field was used to parametrize the proteins, which includes conformational energetics, stretch, bend, and torsion parameters. The potential energy of the proteins was minimized and the simulation was executed in the NPT ensemble to eliminate high energies in the predicted model. The molecular simulation was performed at 1 atm and 300 K for 200 ns. Trajectories were saved for analysis after every 200 ps, and the stability of the simulation was confirmed by comparing the root mean square deviation (RMSD) and root mean square fluctuation (RMSF) of the proteins over time. The trajectory files were also evaluated for radius of gyration (Rg), solvent-accessible surface area (SASA), hydrogen bond analysis, and secondary structure analysis [68].

#### Analysis of the secondary structure

In order to generate the 4 PDB files of average wildtype and mutant structures in 2 segments of the frames after 170 ns, we utilized GROMACS and the gmx covar command with the -av parameter [69]. Subsequently, we performed secondary structure analysis of each structure using STRIDE [70].

#### Principal component analysis (PCA)

Principal component analysis (PCA) is a widely used method for analyzing molecular dynamics (MD) trajectories. This method involves mapping the coordinates of each atom in each frame of the trajectory onto a set of orthogonal vectors (principal components) in order to reduce the dimensionality of the data. The covariance matrix of the atomic coordinates is calculated, and the eigenvalues and eigenvectors are obtained through diagonalization of the covariance matrix, generating the principal components.

In this study, GROMACS software was used to perform PCA. Two commands, gmx covar and gmx anaeig, were utilized for this purpose. Only the coordinates of the C-alpha atoms were considered in the analysis.

#### Free energy landscape (FEL)

The free energy landscape (FEL) is a tool for exploring the energy distribution of a protein in various variables, including principal components, and provides insight into its stability and conformational changes. The Gibbs free energy, which incorporates both enthalpy and entropy, is calculated at each state to generate the FEL.

In our study, FEL analysis was performed using GROMACS with the gmx sham command. The Gibbs free energy was calculated as a function of the first two principal components, considering only the C-alpha atoms.

### Statistical analysis

Using SPSS version 23 and Microsoft Excel, correlation analysis was performed on computational in silico tool predictions. The Student’s t-test was used to investigate significant differences predicted by the numerous computational algorithms. *p* values ≤ 0.001 were considered significant.

## Supplementary Information

**Additional file 1: Figure S1.** Overall significance of the prediction tools used in the study. **Table S1.** Confirmation of the deleterious nsSNPs by other prediction software.

**Additional file 2: File S2.** Results of MutPred2 analysis.

**Additional file 3: File S3.** Consurf analysis.

**Additional file 4: File S4.** Ramachandran Plot for wildtype and mutant model.

**Additional file 5: File S5.** Output from MolProbity all-atom contact analysis.

**Additional file 6: File S6.** The effects of 50 high-risk nsSNPs of ADAMTS3 gene on protein stability predicted by MUpro, INPS3D and MAF.

**Additional file 7: File S7.** Predicted effects of the mutations from 50 high-risk pathogenic nsSNPs of ADAMTS3 on amino acid size, charge and hydrophobicity.

**Additional file 8: File S8.** Changes in 3D protein structures and amino acid structures of important mutations.

**Additional file 9: File S9.**—Phosphorylation analysis of mutant protein by GPS 6.0.

**Additional file 10: File 10.**—Ubiquitylation analysis of mutant protein by BDM-PUB.

**Additional file 11: File 11.**—O-glycosylation analysis of mutant protein by NetOGlyc 4.0.

## Data Availability

All the data analyzed during the study are included with links in the article. The ADAMTS3 missense data was downloaded from https://www.ncbi.nlm.nih.gov/snp.
