# Supplementary material for: In-silico assessment of high-risk non-synonymous SNPs in ADAMTS3 gene associated with Hennekam syndrome and their impact on protein stability and function
Source: BMC Bioinformatics. 2023 Jun 15;24:251. doi: 10.1186/s12859-023-05361-6 (PMC10268432; doi:10.1186/s12859-023-05361-6)
Supplement: Supplementary file 1 — Additional file 1: Figure S1. Overall significance of the prediction tools used in the study. Table S1. Confirmation of the deleterious nsSNPs by other prediction software. [file 12859_2023_5361_MOESM1_ESM.docx]

**Figure 1**: Overall significance of the prediction tools used in the study

**Table 1:** Confirmation of the deleterious nsSNPs by other prediction software.

| **AA Position** | **PROVEAN** | **FATHMM** | **LRT** | **M-CAP** | **MetaSVM** | **MetaLR** | **Mutation Assessor** | **MutationTaster** | **FATHMM-MKL Coding** | **CADD** | **PhD-SNP** | **PANTHER** | **SNPs&GO** | **PON-P2** | **DANN** | **SNAP2** |
| --- | --- | --- | --- | --- | --- | --- | --- | --- | --- | --- | --- | --- | --- | --- | --- | --- |
| G298R | D | D | D | D | D | D | D | D | D | D | D | D | D | D | D | D |
| C567Y | D | D | D | D | D | D | D | D | D | D | D | D | D | D | D | D |
| A370T | D | D | D | D | D | D | D | D | D | D | D | D | D | D | D | D |
| C567R | D | D | D | D | D | D | D | D | D | D | D | D | D | D | D | D |
| G374S | D | D | D | D | D | D | D | D | D | D | N | D | D | D | D | D |
| G983S | D | T | D | D | D | D | D | D | D | D | D | D | D | D | D | D |
| R435H | D | D | D | D | D | D | D | D | D | D | N | D | D | D | D | D |
| Q616H | D | T | D | D | D | D | D | D | D | D | D | D | D | D | D | D |
| I291T | D | T | D | D | D | D | D | D | D | D | D | D | D | D | D | D |
| T668M | D | T | D | D | D | D | D | D | D | D | D | D | D | D | D | D |
| R572C | D | T | D | D | D | T | D | D | D | D | D | D | D | D | D | D |
| R576L | D | T | D | D | D | D | D | D | D | D | D | D | D | D | D | D |
| S58F | D | T | D | D | D | D | D | D | D | D | D | D | N | D | D | D |
| R565W | D | T | D | D | D | T | D | D | D | D | D | D | D | D | D | D |
| A336V | D | D | D | D | D | D | D | D | D | D | N | D | N | D | D | D |
| R959W | D | T | D | D | D | D | D | D | D | D | N | D | D | D | D | D |
| G412S | D | D | D | D | D | D | T | D | D | D | N | D | D | D | D | D |
| P371S | D | D | D | D | D | D | D | D | D | D | N | D | D | U | D | D |
| R883C | D | T | D | D | D | T | D | D | D | D | N | D | D | D | D | D |
| Y636C | D | T | T | D | D | D | D | T | D | D | D | D | D | D | D | D |
| Y536C | D | T | D | D | T | T | D | D | D | D | D | D | D | D | D | D |
| V395I | N | D | D | D | D | D | D | D | D | D | N | D | N | U | D | D |
| R565Q | D | T | D | D | T | T | T | D | D | D | D | D | D | D | D | D |
| S1038F | D | T | D | D | T | T | D | D | D | D | D | D | D | U | D | D |
| D815Y | D | T | D | D | T | T | D | D | D | D | D | D | D | U | D | D |
| L801F | D | T | D | D | T | T | D | D | D | D | D | D | D | U | D | D |
| R954H | D | T | D | D | D | T | D | D | D | D | D | D | D | U | D | D |
| F777L | D | T | D | B | T | T | D | D | D | D | D | D | D | U | D | D |
| R943H | D | T | D | D | T | T | D | D | D | D | N | D | D | U | D | D |
| R94L | D | T | D | D | T | T | D | D | D | D | D | D | N | U | D | D |
| R817C | D | T | D | B | T | T | D | D | D | D | D | D | D | U | D | D |
| R713L | D | T | D | D | T | T | D | D | D | D | D | D | N | U | D | D |
| I287F | D | T | D | D | T | T | D | D | D | D | D | D | D | U | T | D |
| Y148C | D | T | D | B | T | T | D | D | D | D | D | D | D | U | D | D |
| R270H | D | T | D | D | T | T | T | D | D | D | N | D | D | U | D | D |
| M731T | D | T | D | D | T | T | D | D | D | D | N | D | N | D | T | D |
| R248H | D | T | T | D | T | T | D | D | D | D | N | D | D | U | D | D |
| R1053C | D | T | D | D | T | T | T | D | D | D | N | D | D | U | D | D |
| D791V | D | T | D | D | T | T | D | D | D | T | N | D | D | U | D | D |
| R572H | D | T | D | B | T | T | T | D | D | D | N | D | N | D | D | D |
| P513T | D | T | D | B | T | T | T | D | D | D | N | D | N | D | D | D |
| P510A | D | T | D | D | T | T | T | D | D | D | N | D | N | U | D | D |
| F81L | D | T | D | B | T | T | D | D | D | D | N | B | N | N | D | D |
| N98S | D | T | D | B | T | T | D | D | D | D | N | D | N | U | D | N |
| Q927R | D | T | D | D | T | T | T | D | D | T | N | D | N | U | T | D |
| R55L | D | T | T | B | T | T | D | T | D | D | D | B | N | U | D | N |
| Q588H | N | T | D | B | T | T | T | D | D | D | N | D | N | U | D | D |
| G25V | N | T | T | B | T | T | T | D | D | D | N | D | N | U | D | N |
| P77T | N | T | T | B | T | T | T | T | D | T | N | B | N | N | T | N |
| R137W | N | T | T | T | T | T | T | T | T | T | N | B | N | N | T | N |

Threshold: MutationTaster: <0.5 CADD: > 20 MetaLR: > 0.5 M-CAP: > 0.025 PANTHER: probably damaging time > 450my possibly damaging" (450my > time > 200my, "probably benign" (time < 200my). VEST3: > 0.5 LRT: >0.001 PROVEAN: > -2.667 FATHMM-MKK: < 0.5 PHD-SNP: >0.5 SNP-GO: >0.5 SNAP2: −100 (fully neutral) +100 (strong effect) PON-P2: >0.5 DANN: >0.5 Mutation Assessor: > 0.65 (‐5.545 to 5.975 (higher score ‐>  more damaging)) FATHMM: > 0.453
