# Supplementary material for: In-silico assessment of high-risk non-synonymous SNPs in ADAMTS3 gene associated with Hennekam syndrome and their impact on protein stability and function
Source: BMC Bioinformatics. 2023 Jun 15;24:251. doi: 10.1186/s12859-023-05361-6 (PMC10268432; doi:10.1186/s12859-023-05361-6)
Supplement: Supplementary file 3 — Additional file 3: File S3. Consurf analysis. [file 12859_2023_5361_MOESM3_ESM.docx]

**Supplementary File 3 Consurf analysis**


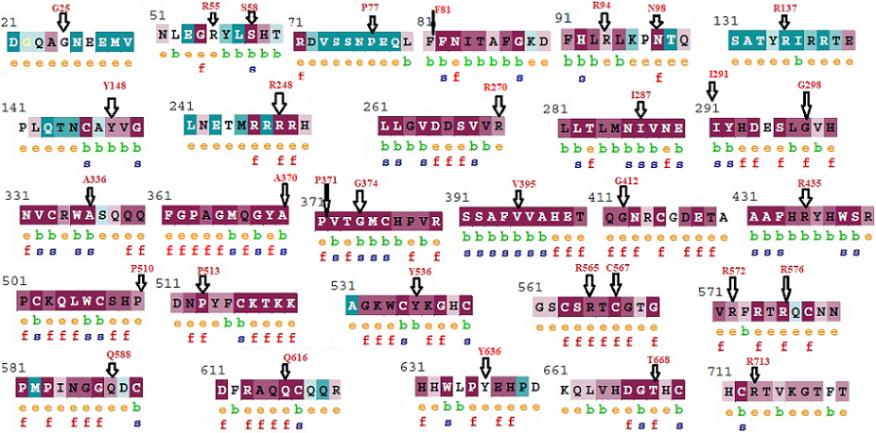


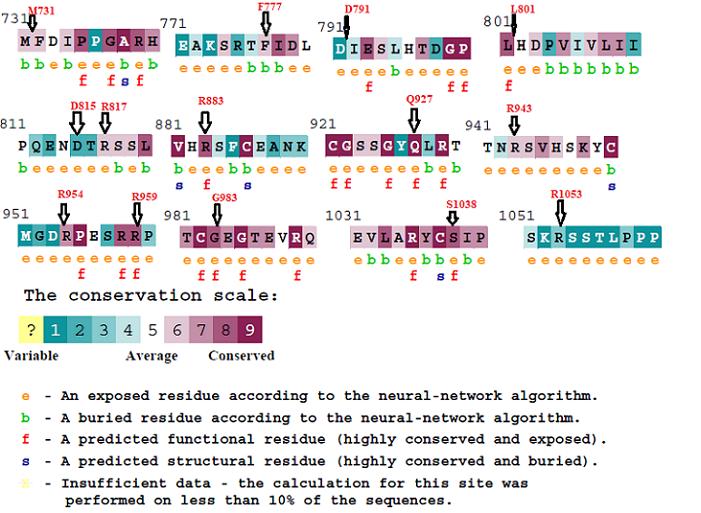


**Supplementary File 3.** The ConSurf server calculated the evolutionary conservation of amino acids in the ADAMTS3 gene. A high variability area is indicated by a value of 1. As the area becomes more conserved, the value rises to a maximum of 9.
