# Supplementary material for: In-silico assessment of high-risk non-synonymous SNPs in ADAMTS3 gene associated with Hennekam syndrome and their impact on protein stability and function
Source: BMC Bioinformatics. 2023 Jun 15;24:251. doi: 10.1186/s12859-023-05361-6 (PMC10268432; doi:10.1186/s12859-023-05361-6)

**Supplementary File 4**: Ramachandran Plot for wildtype and mutant model

Wildtype model


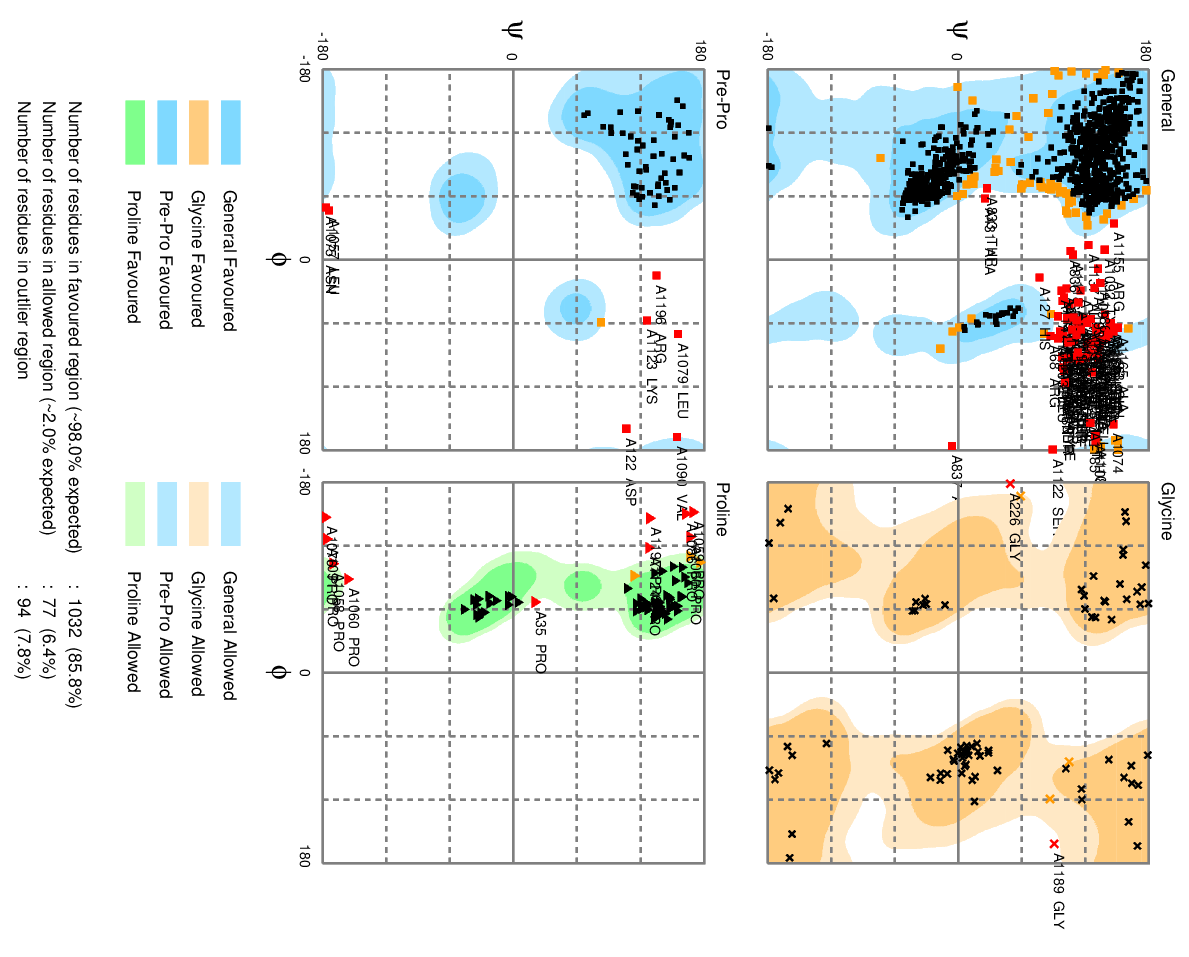


Mutant model


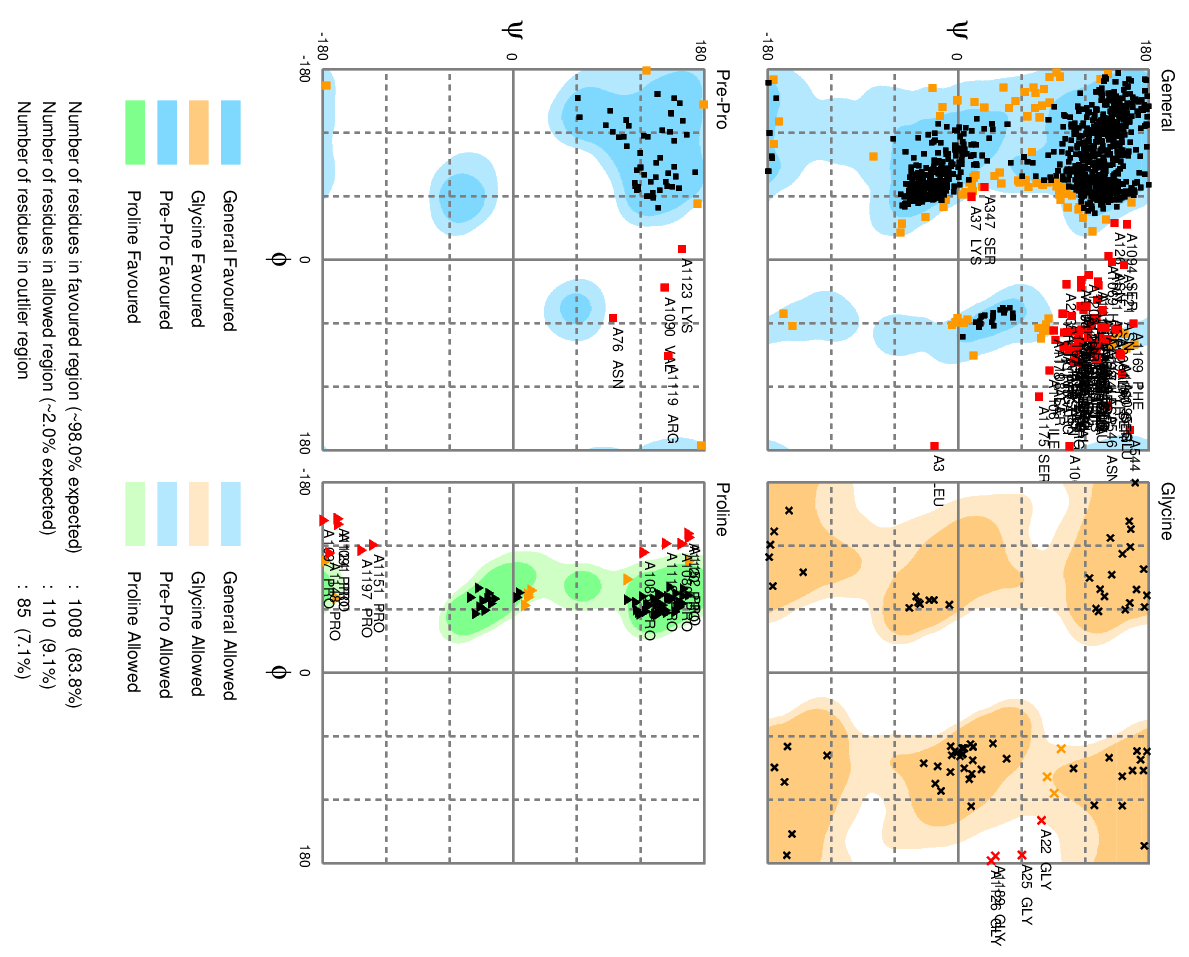

Supplement: Supplementary file 4 — Additional file 4: File S4. Ramachandran Plot for wildtype and mutant model. [file 12859_2023_5361_MOESM4_ESM.docx]
