# Supplementary material for: In-silico assessment of high-risk non-synonymous SNPs in ADAMTS3 gene associated with Hennekam syndrome and their impact on protein stability and function
Source: BMC Bioinformatics. 2023 Jun 15;24:251. doi: 10.1186/s12859-023-05361-6 (PMC10268432; doi:10.1186/s12859-023-05361-6)
Supplement: Supplementary file 5 — Additional file 5: File S5. Output from MolProbity all-atom contact analysis. [file 12859_2023_5361_MOESM5_ESM.docx]

**Supplementary File 5**: Output from MolProbity all-atom contact analysis

Wildtype

Using nuclear cloud x-H distances and vdW radii

No H/D atoms detected - forcing hydrogen addition!

Adding H/D atoms with reduce...

Bad Clashes >= 0.4 Angstrom:

A 731 MET HE2 A 809 ILE HD12 :0.883

A 161 ALA HB2 A 290 GLU HG3 :0.804

A 504 GLN HE22 A 521 GLY N :0.690

A 937 LEU HD11 A 943 ARG HB2 :0.634

A1090 VAL HG13 A1091 PRO CD :0.614

A 422 GLY HA2 A 431 ALA HA :0.576

A 17 ARG O A 18 THR HG23 :0.575

A1168 SER HB3 A1169 PHE CD2 :0.572

A 430 GLN C A 432 ALA H :0.567

A1169 PHE HB3 A1170 PHE CD2 :0.564

A 730 LYS HA A 808 LEU HD23 :0.558

A 142 LEU HD21 A 308 MET HE3 :0.549

A 866 GLN HE21 A 899 CYS HB3 :0.535

A 554 TRP CE2 A 590 CYS HB3 :0.527

A 361 PHE CE2 A 366 MET HE3 :0.518

A1079 LEU HB3 A1080 PRO HD3 :0.515

A 549 LYS HE2 A 551 ASP OD1 :0.514

A 930 THR HG21 A 932 ARG CZ :0.506

A 832 PRO C A 834 ILE H :0.503

A 109 GLU CD A 307 ARG HH21 :0.494

A 948 LYS HE3 A 949 TYR CZ :0.492

A 406 MET HE2 A 446 LEU HB2 :0.492

A 433 PHE C A 434 HIS CG :0.482

A 478 MET SD A 492 MET HE2 :0.470

A 676 TYR CE1 A 689 CYS HA :0.468

A 918 THR HG21 A 926 TYR CE2 :0.465

A 756 LYS HE2 A 761 GLY HA2 :0.459

A1062 LEU HB3 A1063 LEU HD12 :0.455

A1072 VAL HG12 A1073 ILE HD12 :0.449

A1073 ILE HG22 A1074 SER OG :0.448

A1068 THR HA A1069 HIS HB2 :0.447

A 987 GLU CD A1008 ARG HE :0.447

A 43 GLU CD A 87 PHE H :0.443

A 935 GLN HB2 A 945 VAL HG21 :0.441

A1168 SER CB A1169 PHE CD2 :0.438

A 751 HIS HB3 A 809 ILE CG2 :0.438

A 313 TYR CE2 A 317 ILE HD11 :0.436

A 866 GLN NE2 A 899 CYS HB3 :0.435

A1090 VAL HG13 A1091 PRO HD2 :0.435

A 297 LEU HD11 A 446 LEU HD22 :0.430

A 574 ARG HH21 A 596 GLU CD :0.428

A 426 ALA HA A 427 PRO HD3 :0.426

A 34 LEU C A 36 ILE H :0.425

A 493 CYS SG A 508 SER HB3 :0.424

A 84 ILE HD12 A 93 LEU HD11 :0.417

A 549 LYS HE3 A 586 GLY O :0.417

A1072 VAL HG13 A1073 ILE HG13 :0.417

A1173 SER HB3 A1174 ASP CG :0.417

A 533 LYS HE2 A 542 TRP CE2 :0.414

A 649 CYS HB2 A 660 MET HE3 :0.413

A 458 ASP CG A 465 TRP HE1 :0.413

A 571 VAL CG2 A 666 ASP HB3 :0.412

A 142 LEU HD12 A 278 GLN HE21 :0.411

A 713 ARG HH21 A 828 GLU CD :0.411

A 142 LEU CD2 A 308 MET HE3 :0.410

A 161 ALA HB2 A 290 GLU CG :0.409

A 362 GLY HA3 A 366 MET CE :0.408

A 672 TYR CE2 A 778 ILE HD13 :0.407

A 638 HIS CE1 A 643 LYS HD2 :0.406

A 313 TYR CZ A 317 ILE HD11 :0.406

A 366 MET HB3 A 366 MET HE2 :0.405

A 927 GLN HG3 A 961 CYS SG :0.404

A 659 TYR CE1 A 661 LYS HA :0.404

A 751 HIS ND1 A 811 PRO HA :0.404

A 45 VAL HG11 A 86 ALA HB2 :0.403

A 362 GLY HA3 A 366 MET HE1 :0.403

A1072 VAL CG1 A1073 ILE HD12 :0.400

clashscore = 3.61

clashscore (B factor cutoff = 40) = 2.953020

Mutant

Using nuclear cloud x-H distances and vdW radii

No H/D atoms detected - forcing hydrogen addition!

Adding H/D atoms with reduce...

Bad Clashes >= 0.4 Angstrom:

A1167 ALA HB1 A1168 SER HB2 :0.677

A 866 GLN HE21 A 899 CYS HB3 :0.666

A 325 PRO HB3 A 366 MET HE1 :0.625

A1151 PRO HB2 A1152 PRO HD3 :0.592

A 362 GLY HA3 A 366 MET HE2 :0.580

A1177 GLY HA2 A1178 ALA HB3 :0.539

A 553 ASN HA A 590 CYS SG :0.528

A 325 PRO CB A 366 MET HE1 :0.525

A1167 ALA HB1 A1168 SER CB :0.524

A 458 ASP HA A 465 TRP CZ2 :0.514

A 549 LYS HE3 A 587 GLY HA2 :0.510

A 756 LYS HE2 A 761 GLY HA2 :0.490

A 504 GLN HE22 A 521 GLY N :0.471

A1166 ALA HB1 A1167 ALA HB2 :0.470

A 582 MET HG3 A 583 PRO HD2 :0.470

A1164 MET HB3 A1165 ALA HB2 :0.469

A1197 PRO HB2 A1198 THR HG23 :0.463

A1151 PRO CB A1152 PRO HD3 :0.453

A1073 ILE HG23 A1074 SER H :0.450

A 673 LYS HE3 A 783 GLU OE1 :0.439

A 346 HIS C A 348 GLU H :0.435

A 676 TYR CE1 A 689 CYS HA :0.432

A 550 GLN O A 587 GLY HA3 :0.431

A1178 ALA HA A1179 SER HB2 :0.428

A 259 GLU HB2 A 351 ASP H :0.426

A 832 PRO C A 834 ILE H :0.424

A1198 THR HG22 A1199 ARG CB :0.423

A1101 MET HA A1102 SER CB :0.420

A1147 VAL HG12 A1148 PRO HD3 :0.416

A 755 ILE HG22 A 764 ILE HD12 :0.416

A1101 MET HA A1102 SER HB3 :0.413

A1198 THR HG22 A1199 ARG HB3 :0.408

A 730 LYS HA A 808 LEU HD23 :0.407

A 582 MET CG A 583 PRO HD2 :0.407

A 34 LEU C A 36 ILE H :0.405

clashscore = 1.88

clashscore (B factor cutoff = 40) = 2.897024
