# Supplementary material for: In-silico assessment of high-risk non-synonymous SNPs in ADAMTS3 gene associated with Hennekam syndrome and their impact on protein stability and function
Source: BMC Bioinformatics. 2023 Jun 15;24:251. doi: 10.1186/s12859-023-05361-6 (PMC10268432; doi:10.1186/s12859-023-05361-6)
Supplement: Supplementary file 7 — Additional file 7: File S7. Predicted effects of the mutations from 50 high-risk pathogenic nsSNPs of ADAMTS3 on amino acid size, charge and hydrophobicity. [file 12859_2023_5361_MOESM7_ESM.docx]

Supplementary File 7. Predicted effects of the mutations from 50 high-risk pathogenic nsSNPs of ADAMTS3 on amino acid size, charge and hydrophobicity.

| Mutation | ConSurf  Conservation score | Amino acids size | Charge | Hydrophobicity |
| --- | --- | --- | --- | --- |
| G298R | 9 | M>W | - |  |
| C567Y | 9 | W>M | - |  |
| A370T | 8 | W<M | Neu→Neg | ↓ |
| C567R | 8 | W<M | - |  |
| G374S | 9 | W<M | Neg→Neu |  |
| G983S | 9 | W<M | Neg→Neu | ↑ |
| R435H | 8 | W<M | Neu→Pos | ↓ |
| Q616H | 8 | W<M | Neu→Neg | ↓ |
| I291T | 9 | W>M |  | ↓ |
| T668M | 9 | W<M | Neg→Pos |  |
| R572C | 9 | W<M | Neu→Pos |  |
| R576L | 9 | W<M |  | ↑ |
| S58F | 9 | W>M |  |  |
| R565W | 8 | W<M |  |  |
| A336V | 9 | W>M |  |  |
| R959W | 9 | W<M |  |  |
| G412S | 9 | W>M |  |  |
| P371S | 9 | W>M | Pos→Neu |  |
| R883C | 9 | W>M | Pos→Neu | ↑ |
| Y636C | 9 | W>M | Pos→Neu | ↑ |
| Y536C | Transmembrane 1 | W<M |  | ↑ |
| V395I | Transmembrane 1 | W<M |  |  |
| R565Q | Transmembrane 1 | W>M | Neg→Neu | ↑ |
| S1038F | 9 | W<M |  |  |
| D815Y | 9 | W<M |  |  |
| L801F | 9 | W>M |  | ↓ |
| R954H | 9 |  | Neg→Neu |  |
| F777L | 9 | W<M | Neg→Neu | ↑ |
| R943H | 9 | W>M | Neg→Neu | ↑ |
| R94L | 9 | W<M |  | ↑ |
| R817C | 9 |  | Neu→Neg |  |
| R713L | 9 | W<M |  |  |
| I287F | 7 | W<M | Neg→Pos |  |
| Y148C | 9 | W>M |  | ↑ |
| R270H | 9 |  | Neg→Neu |  |
| M731T | 9 | W>M | Pos→Neu | ↑ |
| R248H | 9 | W>M | Pos→Neu |  |
| R1053C | 8 | W>M |  |  |
| D791V | 9 | W>M |  | ↑ |
| R572H | 9 | W<M |  | ↓ |
| P513T | 9 | W<M |  |  |
| P510A | 9 | W<M |  |  |
| F81L | 9 | W>M |  | ↑ |
| N98S | 9 | W<M |  | ↑ |
| Q927R | 9 | W<M |  |  |
| R55L | 9 | W>M |  | ↓ |
| Q588H | 8 | W<M |  |  |
| G25V | 9 | W<M |  | ↑ |
| P77T | 9 | W>M |  |  |
| R137W | 8 | W>M | Pos→Neu | ↑ |
