# Supplementary material for: In-silico assessment of high-risk non-synonymous SNPs in ADAMTS3 gene associated with Hennekam syndrome and their impact on protein stability and function
Source: BMC Bioinformatics. 2023 Jun 15;24:251. doi: 10.1186/s12859-023-05361-6 (PMC10268432; doi:10.1186/s12859-023-05361-6)
Supplement: Supplementary file 8 — Additional file 8: File S8. Changes in 3D protein structures and amino acid structures of important mutations. [file 12859_2023_5361_MOESM8_ESM.docx]

**Supplementary File 8**: Changes in 3D protein structures and amino acid structures of important mutations

|  |  | Wildtype | Mutant |
| --- | --- | --- | --- |
| S58F | 3D protein structures | 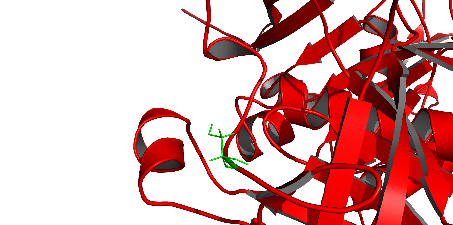 | 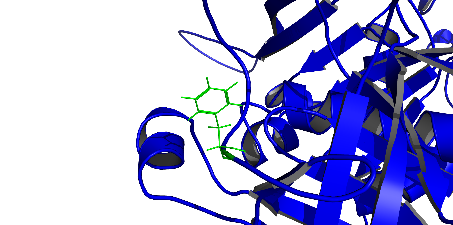 |
|  | Amino acid structures | 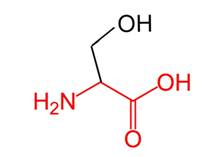 | 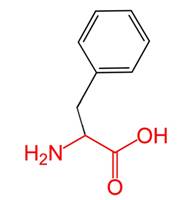 |
| R138K | 3D protein structures | 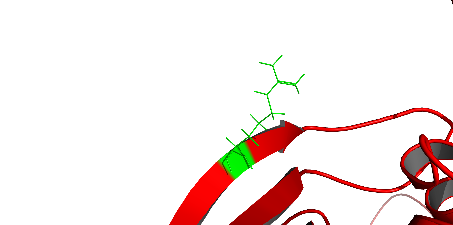 | 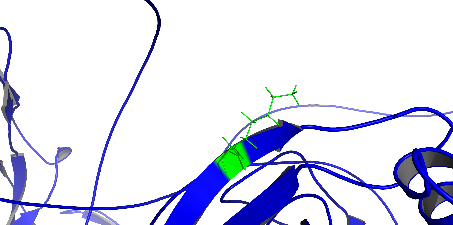 |
|  | Amino acid structures | 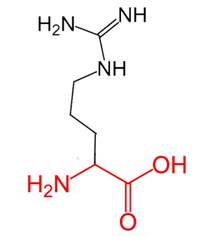 | 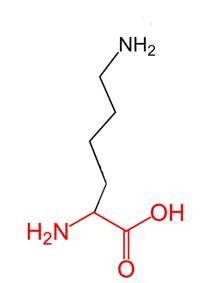 |
| I291T | 3D protein structures | 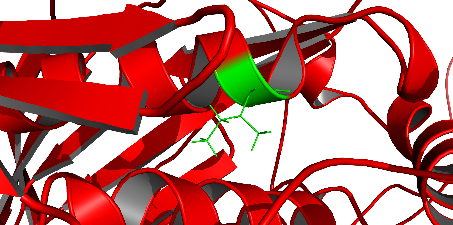 | 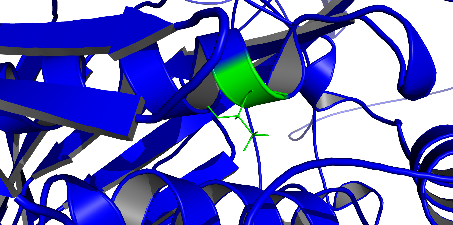 |
|  | Amino acid structures | 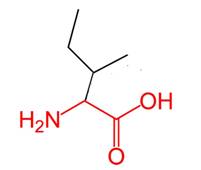 | 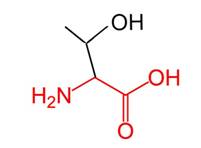 |
| A336V | 3D protein structures | 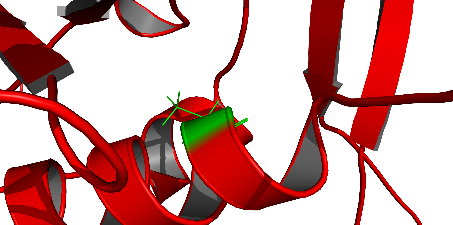 | 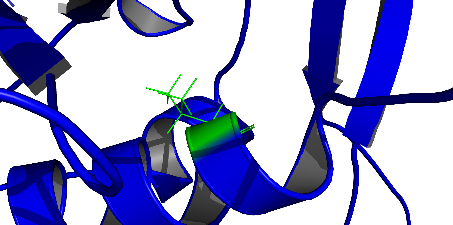 |
|  | Amino acid structures | 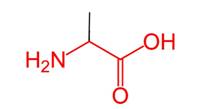 | 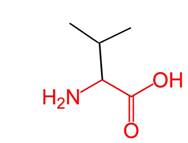 |
| P371S | 3D protein structures | 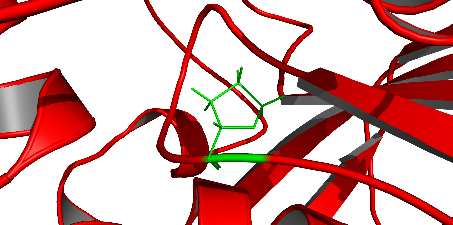 | 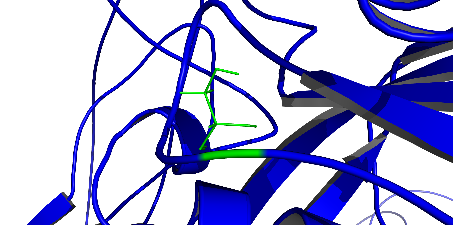 |
|  | Amino acid structures | 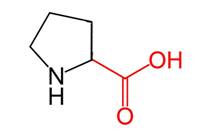 | 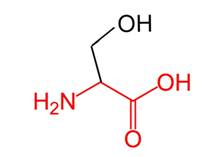 |
| G412S | 3D protein structures | 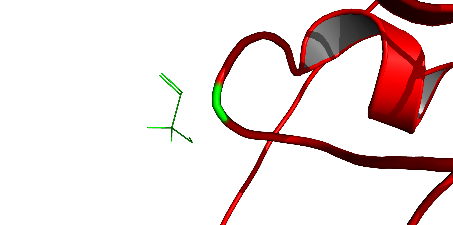 | 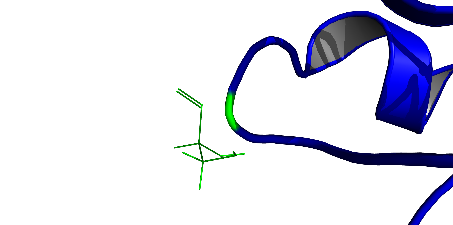 |
|  | Amino acid structures | 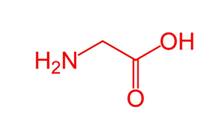 | 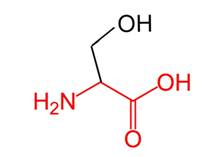 |
| R435H | 3D protein structures | 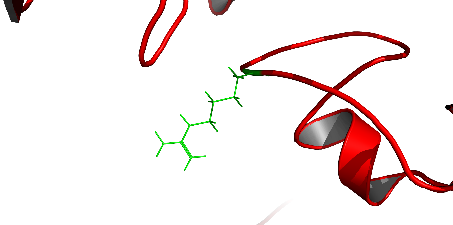 | 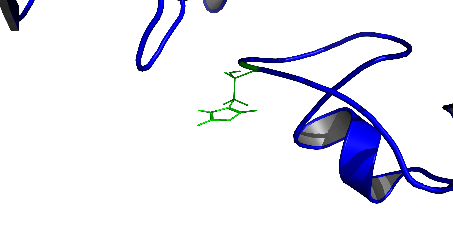 |
|  | Amino acid structures | 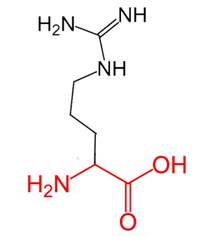 | 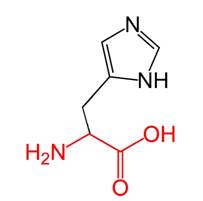 |
| R565W | 3D protein structures | 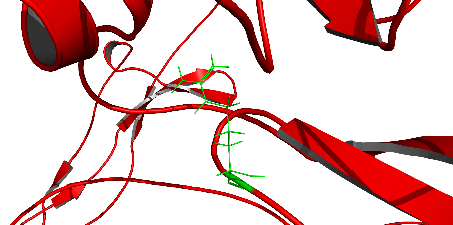 | 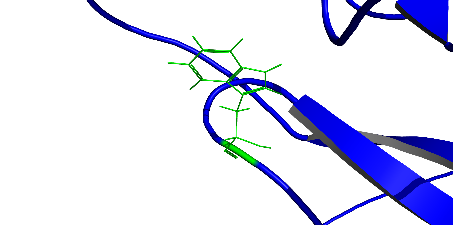 |
|  | Amino acid structures | 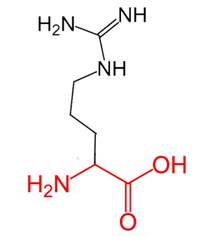 | 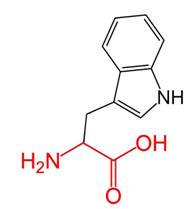 |
| R572C | 3D protein structures | 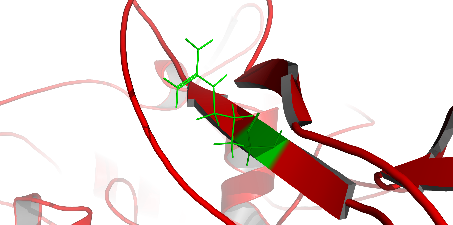 | 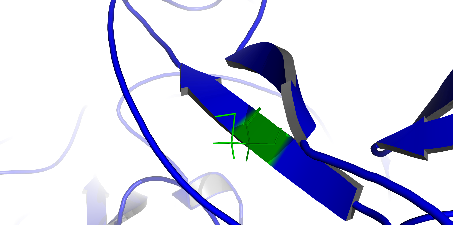 |
|  | Amino acid structures | 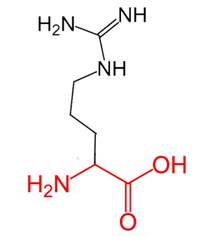 | 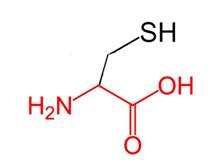 |
| R574C | 3D protein structures | 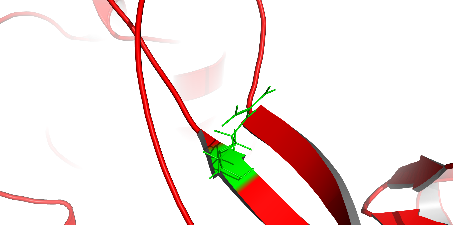 | 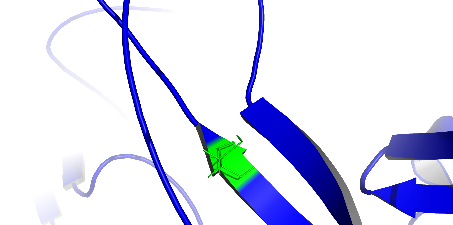 |
|  | Amino acid structures | 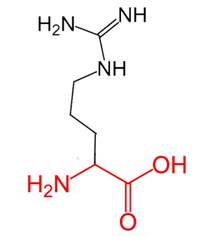 | 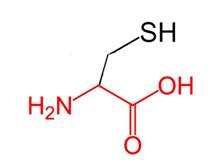 |
| R576L | 3D protein structures | 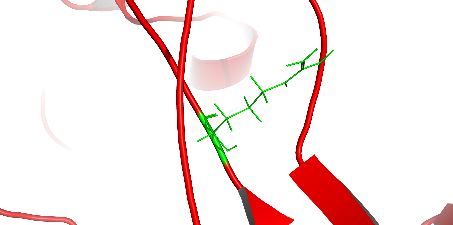 | 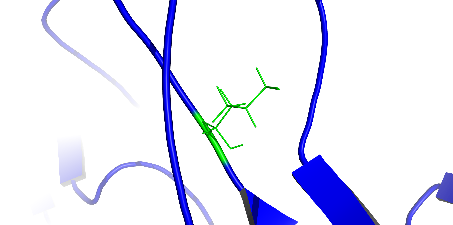 |
|  | Amino acid structures | 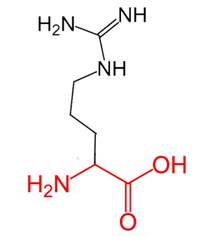 | 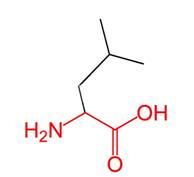 |
| C578L | 3D protein structures | 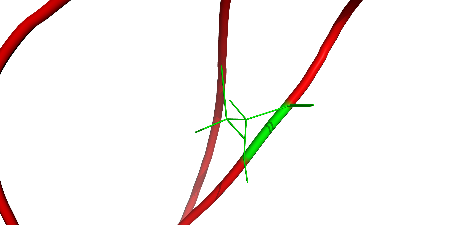 | 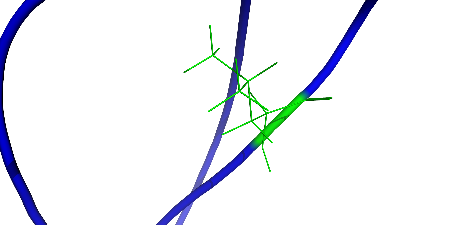 |
|  | Amino acid structures | 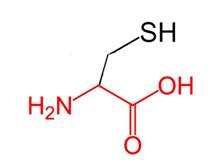 | 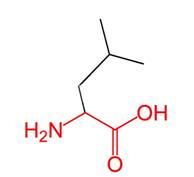 |
| Q606H | 3D protein structures | 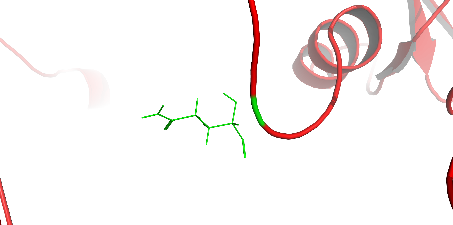 | 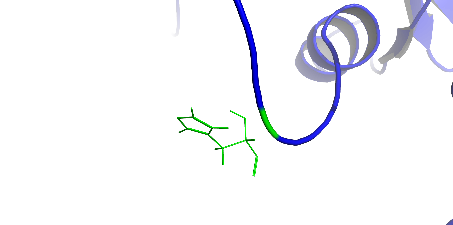 |
|  | Amino acid structures | 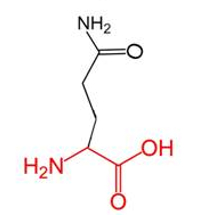 | 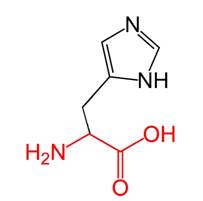 |
| Q616H | 3D protein structures | 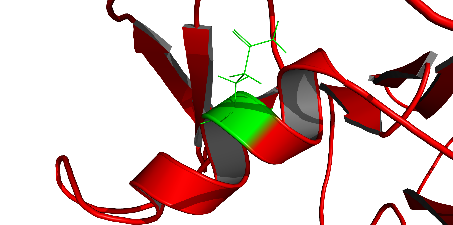 | 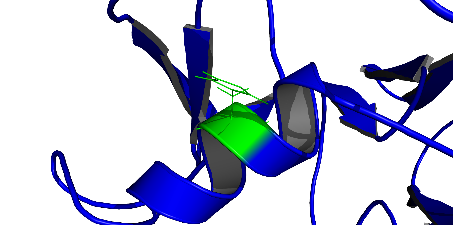 |
|  | Amino acid structures | 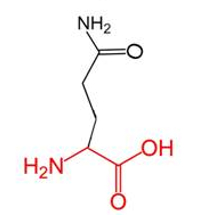 | 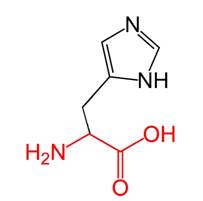 |
| Y636C | 3D protein structures | 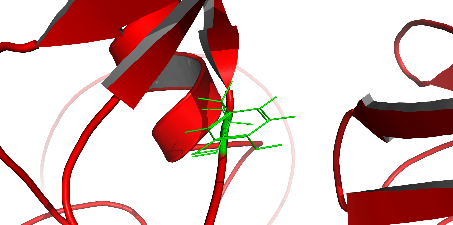 | 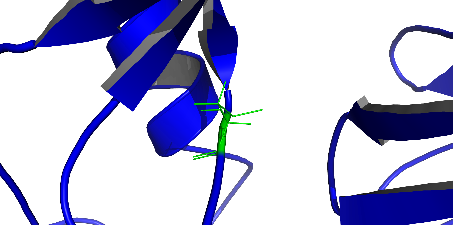 |
|  | Amino acid structures | 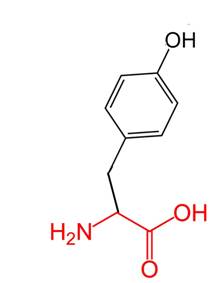 | 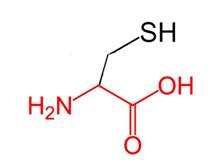 |
| T668M | 3D protein structures | 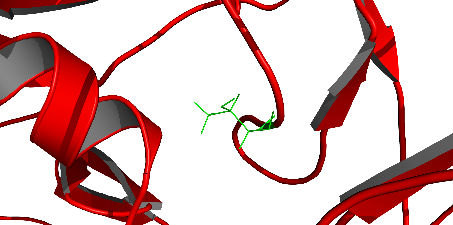 | 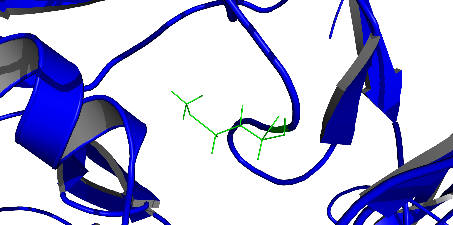 |
|  | Amino acid structures | 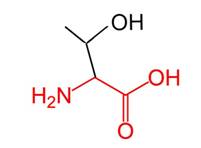 | 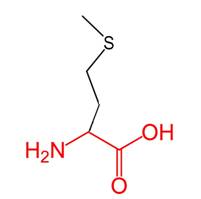 |
| R883C | 3D protein structures | 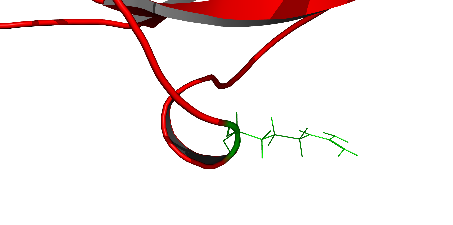 | 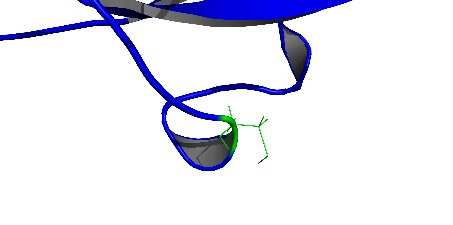 |
|  | Amino acid structures | 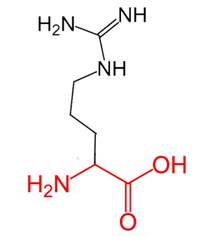 | 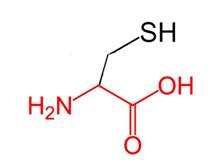 |
| R954H | 3D protein structures | 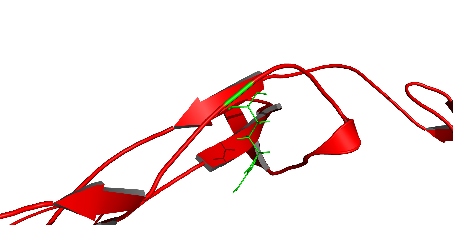 | 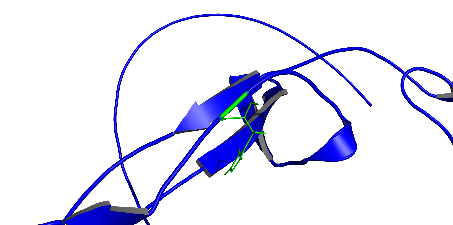 |
|  | Amino acid structures | 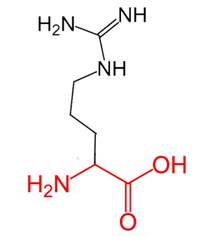 | 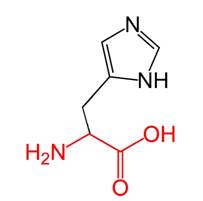 |
| R959W | 3D protein structures | 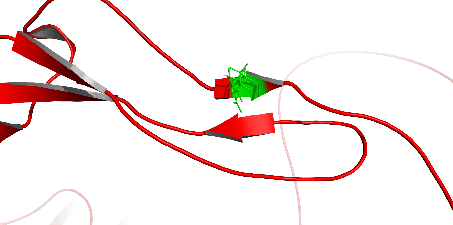 | 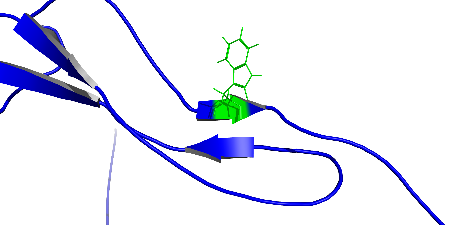 |
|  | Amino acid structures | 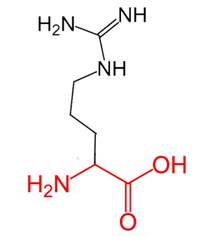 | 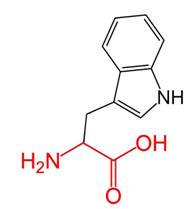 |
| G983S | 3D protein structures | 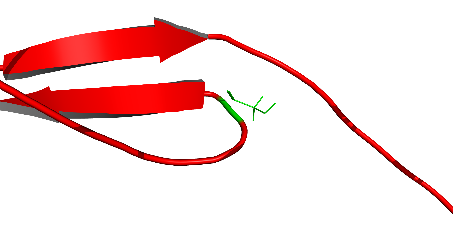 | 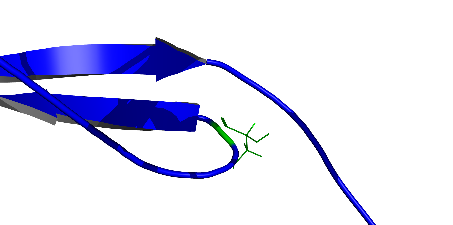 |
|  | Amino acid structures | 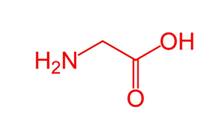 | 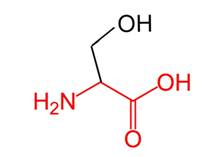 |
